# A host receptor enables type 1 pilus-mediated pathogenesis of *Escherichia coli* pyelonephritis

**DOI:** 10.1371/journal.ppat.1009314

**Published:** 2021-01-29

**Authors:** Lisa K. McLellan, Michael R. McAllaster, Arthur S. Kim, Ľubomíra Tóthová, Patrick D. Olson, Jerome S. Pinkner, Allyssa L. Daugherty, Teri N. Hreha, James W. Janetka, Daved H. Fremont, Scott J. Hultgren, Herbert W. Virgin, David A. Hunstad

**Affiliations:** 1 Department of Pediatrics, Washington University School of Medicine, St. Louis, Missouri, United States of America; 2 Department of Pathology and Immunology, Washington University School of Medicine, St. Louis, Missouri, United States of America; 3 Department of Medicine, Washington University School of Medicine, St. Louis, Missouri, United States of America; 4 Department of Molecular Microbiology, Washington University School of Medicine, St. Louis, Missouri, United States of America; 5 Department of Biochemistry and Molecular Biophysics, Washington University School of Medicine, St. Louis, Missouri, United States of America; University of Utah, UNITED STATES

## Abstract

Type 1 pili have long been considered the major virulence factor enabling colonization of the urinary bladder by uropathogenic *Escherichia coli* (UPEC). The molecular pathogenesis of pyelonephritis is less well characterized, due to previous limitations in preclinical modeling of kidney infection. Here, we demonstrate in a recently developed mouse model that beyond bladder infection, type 1 pili also are critical for establishment of ascending pyelonephritis. Bacterial mutants lacking the type 1 pilus adhesin (FimH) were unable to establish kidney infection in male C3H/HeN mice. We developed an *in vitro* model of FimH-dependent UPEC binding to renal collecting duct cells, and performed a CRISPR screen in these cells, identifying desmoglein-2 as a primary renal epithelial receptor for FimH. The mannosylated extracellular domain of human DSG2 bound directly to the lectin domain of FimH *in vitro*, and introduction of a mutation in the FimH mannose-binding pocket abolished binding to DSG2. In infected C3H/HeN mice, type 1-piliated UPEC and Dsg2 were co-localized within collecting ducts, and administration of mannoside FIM1033, a potent small-molecule inhibitor of FimH, significantly attenuated bacterial loads in pyelonephritis. Our results broaden the biological importance of FimH, specify the first renal FimH receptor, and indicate that FimH-targeted therapeutics will also have application in pyelonephritis.

## Author summary

Urinary tract infections (UTIs) are among the most common bacterial infections in humans. While much has been discovered about how *E*. *coli* cause bladder infections, less is known about the host-pathogen interactions that underlie kidney infection (pyelonephritis). We employed recently developed mouse models to show that bacterial surface fibers called type 1 pili, which bear the adhesive protein FimH and are known to mediate *E*. *coli* binding to bladder epithelium, are also required for ascending kidney infection. We developed a cell-culture model of bacterial binding to renal collecting duct, then performed a screen using the gene-editing tool CRISPR to identify the first known FimH receptor in the kidney. This epithelial cell-surface protein, desmoglein-2, was shown to directly bind FimH, and we localized this binding to specific extracellular domains of DSG2. Further, we showed that mannosides, small-molecule FimH inhibitors currently in development to treat bladder infection, are also effective in experimental kidney infection. Our study reveals a novel host-pathogen interaction during pyelonephritis and demonstrates how this interaction may be therapeutically targeted.

## Introduction

Bacterial adhesion to urinary tract epithelium is a critical step in establishing urinary tract infection (UTI). Strains of uropathogenic *Escherichia coli* (UPEC), the main causative agents of UTIs, carry in their genomes a variable repertoire of adhesive pili assembled via the canonical chaperone-usher pathway (CUP) [1,2]. Given their importance in host-pathogen interactions, UPEC adhesins including CUP pili have received considerable attention as mediators of pathogenesis and potential therapeutic targets [2–5]. Within the mammalian bladder, UPEC employ type 1 pili terminating in the FimH adhesin, which binds α-d-mannose with stereochemical specificity. FimH-mediated UPEC binding to mannosylated uroplakins that coat the luminal surfaces of superficial uroepithelial (facet) cells is a key event in the pathogenesis of cystitis [6–11]. UPEC can subsequently be internalized into facet cells, where they replicate and establish intracellular bacterial communities (IBCs) [12–16]. Bacteria within IBCs are protected from phagocytosis and other host defenses, and this community behavior within the bladder is a critical component of UPEC cystitis [17,18].

Less is known about the host-pathogen interactions enabling establishment of upper-tract UTIs, including pyelonephritis and renal abscess. Another CUP pilus, the P pilus, is tipped with the PapG adhesin, which has been implicated in human pyelonephritis [2,19–21]. However, data regarding the participation of P pili in murine pyelonephritis vary among prior studies, potentially due to disparities in host and bacterial strains used, and species specificity in the glycolipid receptors for PapG [21,22]. Preclinical modeling of UTIs has been performed predominantly in female mice, and in a majority of murine backgrounds, upper-tract UTI resolves spontaneously and without significant sequelae [23,24]. Historically, female mice have been utilized in models of experimental UTI, typically initiated by catheter-directed inoculation of the mouse bladder; reliable catheter access to the male mouse bladder is technically challenging [25–28]. We previously developed an inoculation technique for initiating UTI in both male and female mice; when normal anatomic protections in males were thus bypassed, males evidenced more severe UTI, reflected throughout the course of infection by higher bladder and kidney bacterial loads, leukocyte infiltration, and inflammation [29]. Of note, these findings aligned with human epidemiologic data showing higher morbidity from complicated UTI in men, and higher UTI incidence in women with polycystic ovary syndrome (a common hyperandrogenic state) [30–36]. In C3H/HeN mice (a background inherently featuring vesicoureteral reflux, a primary risk factor for upper-tract UTI in humans), males and androgen-exposed females developed severe pyelonephritis and renal abscesses [29,37]. Renal infection was nucleated by collections of UPEC occupying collecting ducts and more proximal segments of the nephron, which we termed kidney bacterial communities (KBCs) [37].

The consistent development of severe pyelonephritis in this model enables detailed investigation of bacterial and host factors involved in the establishment of ascending upper-tract UTI. We identified a previously unappreciated role in the kidney for the mannose-binding type 1 pilus adhesin FimH, previously recognized as a major urovirulence factor within the bladder. High-affinity mannosides, which neutralize FimH function and are in development for the treatment of cystitis [4,5,38,39], here significantly lowered bacterial burdens in the kidneys of mice with established pyelonephritis. To specify host factors required for upper-tract UTI, we performed a CRISPR-Cas9 screen in immortalized murine renal epithelial cells and identified desmoglein-2 (Dsg2) as a candidate receptor for FimH. The lectin domain of FimH (but not FimH with a point mutation that abolishes mannose binding) directly bound to the extracellular domain of human desmoglein-2 (DSG2). Finally, we observed co-localization of UPEC with Dsg2 in the collecting ducts of mice with ascending pyelonephritis. Our studies demonstrate that disruption of FimH binding to renal tubular epithelium represents a potential therapeutic intervention during pyelonephritis.

## Results

### Type 1 pili are required for both bladder and kidney infection in male C3H/HeN mice

**Fig 1:**
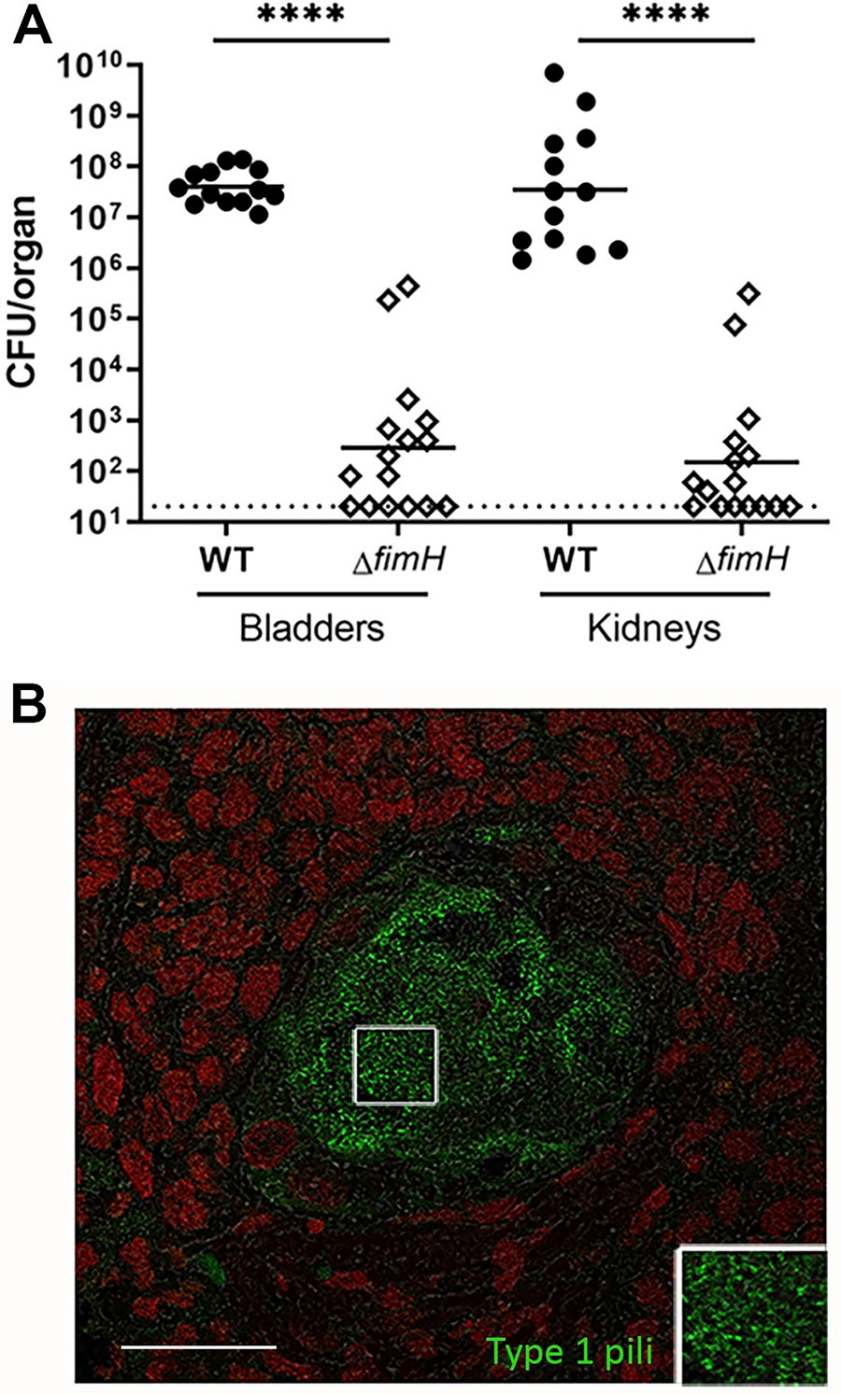
Type 1 pili are required for bladder and kidney infection. **A**) Male C3H/HeN mice were infected with UTI89 (closed circles) or UTI89Δ*fimH* (open diamonds), and organs were harvested 2 wpi. Bacterial loads after WT infection were significantly higher than with Δ*fimH* in both the bladder and kidneys (****p<0.0001). Horizontal bars indicate geometric mean, and dotted line indicates limit of detection. n = 13–16 mice per group over 3 independent experiments. **B**) Immunofluorescence microscopy of C3H/HeN male kidney 2 wpi with UTI89, showing expression of type 1 pili within kidney bacterial communities (magnified in inset; green, anti-type 1 pili; red, SYTO 61 nuclear stain). Scale bar, 20 μm.

Type 1 pili have long been implicated in pathogenesis of UPEC cystitis, but only recently have new experimental models enabled molecular interrogation of their role in pyelonephritis. We infected male C3H/HeN mice with either wild-type UTI89 (a prototypic UPEC strain), or UTI89Δ*fimH*, an isogenic mutant lacking the type 1 pilus adhesin [16,40]. Two weeks post infection (wpi), UTI89Δ*fimH* was sharply attenuated compared to wild-type in bladder and kidney bacterial loads (p<0.0001; **Fig 1A**) and in incidence of visible renal abscess (1/16 *vs* 13/13; p<0.0001). We confirmed that UTI89Δ*fimH* reached the kidney normally following bladder inoculation, as kidney bacterial loads 24 hours post infection (hpi) were equivalent to wild-type (**S1 Fig**). Of note, an analogous defect was confirmed using the urosepsis UPEC isolate CFT073, whose isogenic CFT073Δ*fimH* mutant failed to colonize the kidney 2 wpi (**S2 Fig**). Using immunofluorescence microscopy, we demonstrated type 1 pili expression by UPEC in KBCs located within renal abscesses 2 wpi (**Fig 1B**). As UTI89 carries ten full or partial CUP operons [41], we similarly tested deletion mutants in each of the annotated CUP pili. No other CUP pilus mutants exhibited a defect in bladder or kidney infection in male C3H mice 2 wpi, after inoculation either alone (**S3 Fig**) or in competition with antibiotic-tagged UTI89 (**S4 Fig**).

The pilin and lectin domains of FimH together exist in an equilibrium between tense and relaxed states with differential mannose binding affinity. Conversion between these states is governed in part by a catch-bond mechanism [9,42–44]. In the relaxed state, the FimH lectin domain samples conformational ensembles, allowing it to act as a molecular tether, with the mannose binding pocket in a conformation able to bind mannose tightly; in the tense state, the pocket is open and thus binds mannose only weakly [8]. In the bladder, the ability of FimH to transition between these states enables optimal epithelial binding by UPEC [8,45,46]. We infected male C3H/HeN mice with wild-type UTI89 or with UTI89Δ*fimH* complemented with FimH_A27V/V163A_, a variant that predominantly adopts the relaxed conformation (high-affinity mannose-binding state). Paradoxically, UPEC expressing this variant are attenuated during cystitis in female mice [8,45,46]. Here, we found similarly that UTI89 FimH_A27V/V163A_ also failed to establish pyelonephritis, with significantly lower bacterial loads in kidneys (p = 0.001) as well as bladder (p<0.0001) compared to wild-type UTI89 (**S5 Fig**). Further, the type 1 pilus rod, formed from FimA subunits, normally can unwind from a tightly coiled helix to a more linearized rod; this action is hypothesized to help UPEC withstand the shear force of urine flow [47–49]. A variant in the type 1 pilus helical rod, FimA_A22R_, requires less force to adopt the unwound form, but UPEC expressing this variant are attenuated in the female mouse bladder [50]. We found that UTI89 expressing FimA_A22R_ was also attenuated in the kidneys (p = 0.0038) and bladders (p<0.0001) of male C3H/HeN mice 2 wpi (**S5 Fig**). Thus, the conformational dynamics of type 1 pili, critical in establishing bladder infection, are similarly important in ascending pyelonephritis. Collectively, our data demonstrate that beyond their well-described role in establishing bladder infection, UPEC type 1 pili are also important in initiating ascending pyelonephritis and renal abscess.

### Murine collecting duct cells display type 1 pili-dependent UPEC binding

**Fig 2:**
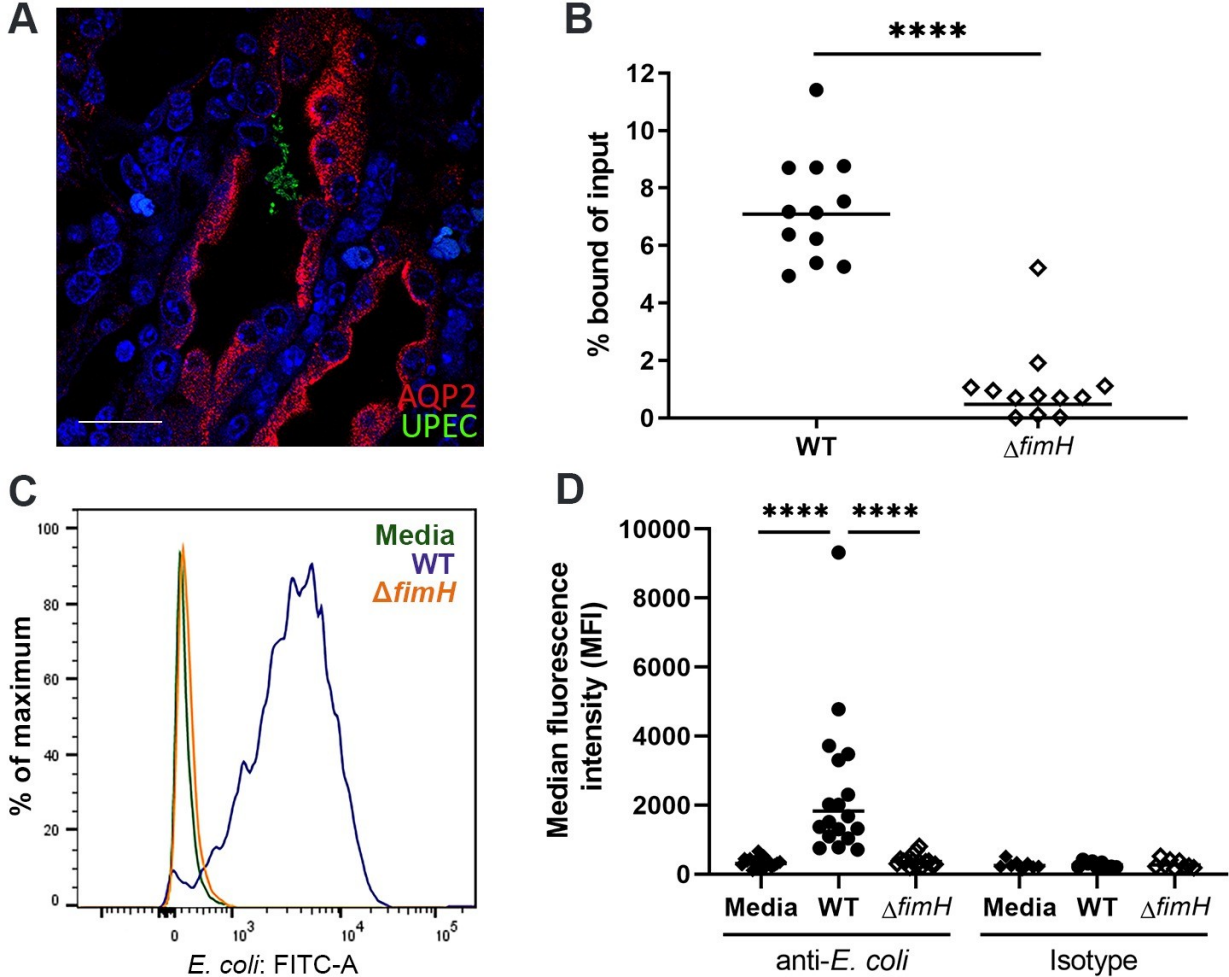
Murine collecting duct cells display type 1 pili-dependent UPEC binding. **A**) Early intratubular colonies of UPEC (green) were localized 5 dpi in male C3H/HeN mice collecting duct tubules (AQP2+, red; DAPI, blue; scale bar, 10 μm). **B**) To intramedullary collecting duct (IMCD-3) cells, WT UTI89 (filled circles) displayed significantly higher levels of bacterial binding than Δ*fimH* (open diamonds; ****p<0.0001) (MOI 20). Horizontal bars indicate geometric mean. n = 12 samples per group over 4 independent experiments. **C, D**) Binding of IMCD-3 cells by UTI89 or Δ*fimH* was quantified by flow cytometry after gating on single cells. **C**) FITC signal (anti-*E*. *coli*) was evident in IMCD-3 cells infected with wild-type UTI89 (navy blue) compared with medium alone (green); infection with Δ*fimH* yielded negligible signal (orange). **D**) Cells infected with UTI89 displayed significantly higher median fluorescence intensity (MFI) than those treated with medium alone or Δ*fimH* (****p<0.0001). n = 8–15 samples per group over 6 independent experiments.

The collecting duct is the first nephron segment encountered by ascending UPEC. Consistent with prior findings [51,52], ascending UPEC were located within collecting ducts in C3H/HeN mice 5 dpi (**Fig 2A**). Therefore, we chose mouse intramedullary collecting duct (IMCD-3) cells to establish an *in vitro* model of type 1 pili binding to kidney epithelium. In standard bacterial binding assays, UTI89 Δ*fimH* was significantly attenuated compared to wild-type UTI89 (**Fig 2B**), recapitulating what we observed in *in vivo* infection. Following IMCD-3 cell infection and anti-*E*. *coli* antibody staining, we confirmed by flow cytometry the *in vitro* binding defect of Δ*fimH* (**Fig 2C and 2D**). This defect was complemented by chromosomal re-integration of wild-type *fimH*, but not by integration of the *fimH*^Q133K^ mutant, which lacks mannose binding activity [10] (**S6 Fig**). The binding defect was also replicated in CFT073 Δ*fimH* (**S6 Fig**). Finally, UPEC binding to collecting duct cells was significantly inhibited by methyl α-d-mannopyranoside and to an even greater extent by mannoside FIM1033 (**S7 Fig**). Thus, the *in vivo* requirement for type 1 pilus function is reflected in *in vitro* binding of UPEC to IMCD-3 cells.

### A CRISPR screen identifies candidate type 1 pilus receptor desmoglein-2

**Fig 3:**
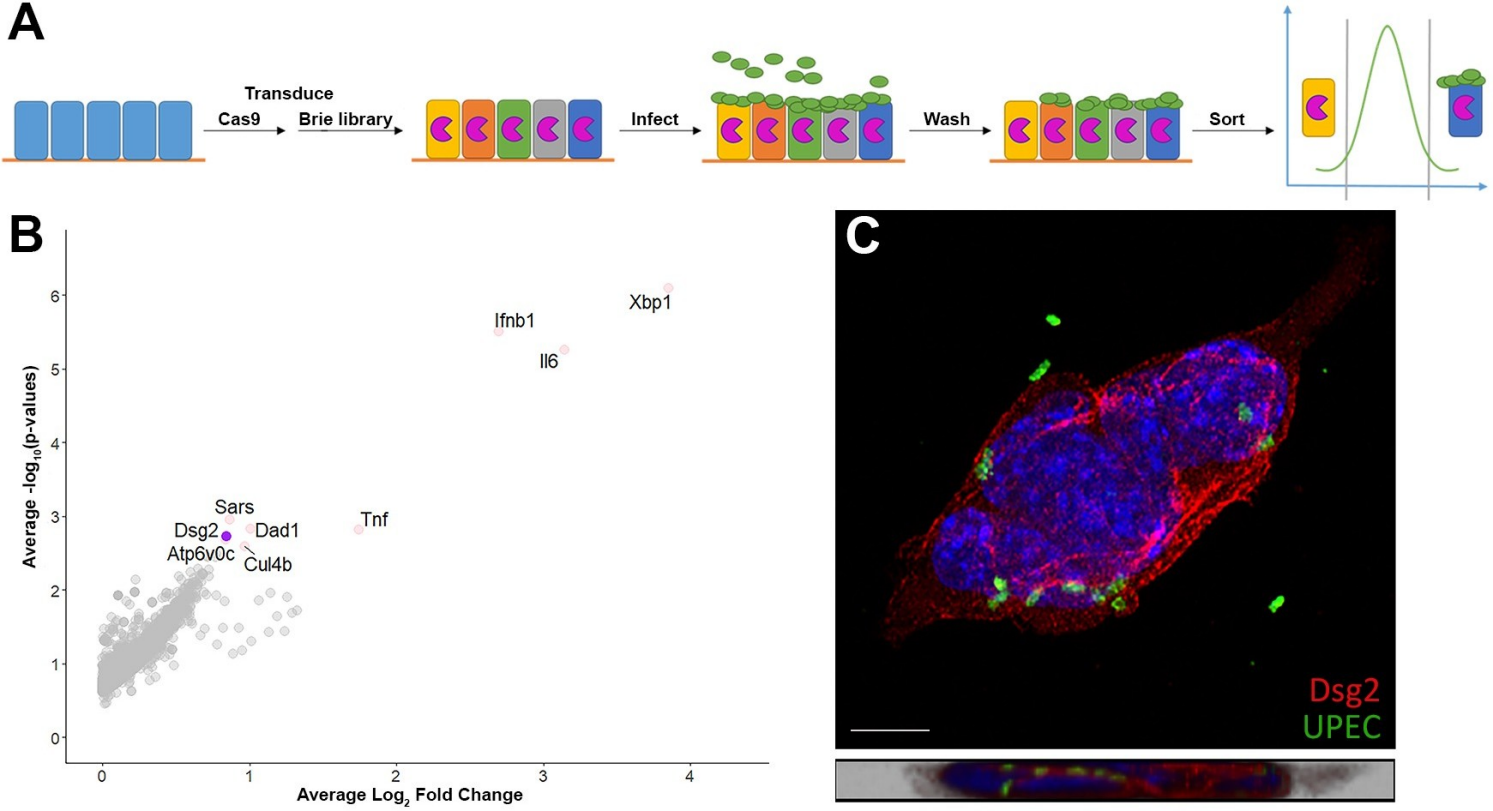
A lentiviral CRISPR screen identifies candidate type 1 pilus receptor desmoglein-2. **A**) Schematic of screen design. To screen for host genes responsible for type 1 pili-dependent binding to this cell line, we transduced the Brie library of mouse guide RNAs into IMCD-3 cells bearing Cas9, providing 4× nominal coverage of each gene within the mouse genome. Cells were then bound (MOI 150) by UTI89 and sorted by fluorescent labeling, isolating cells unbound by bacteria. Genomic DNA was extracted and sequenced, identifying candidate genes that may be required for UPEC binding to IMCD-3 cells. **B**) Volcano plot results from sorted and sequenced cells. Colored dots represent genes having an average log_2_ fold change >0.5 and a–log_10_(p-value) >2.5. **C**) After *in vitro* binding, desmoglein-2 (red) on IMCD-3 cells co-localized with UPEC (green); scale bar, 5 μm. Lower panel demonstrates co-localization in orthogonal view of z-stack projection.

Although type 1 pili are well known as critical virulence factors in the bladder [6–11], they have not been implicated in ascending pyelonephritis, and a cognate receptor has not been identified. To screen for host genes participating in type 1 pili-dependent binding of UPEC to collecting duct epithelium, we performed a genome-wide CRISPR-Cas9 screen using the Brie sgRNA library in IMCD-3 cells (**Fig 3A**), providing 4× nominal coverage of each gene within the mouse genome [53]. The pooled library of edited cells were inoculated with UPEC, fixed and stained, and subsequently flow-sorted for the population of cells with the lowest fluorescence (**Fig 3A**). Genomic DNA was extracted from sorted cells, subjected to Illumina sequencing, and analyzed using the probability mass function of a hypergeometric distribution to identify candidate genes statistically associated with bacterial binding (**S1 Table**) [53,54]. Among these candidates (**Fig 3B**), we focused on genes encoding proteins that would localize to the cell surface and could be available to interact with bacterial pili [55–57]. The screen revealed deficient UPEC binding to cells edited with guides targeting desmoglein-2 (*Dsg2*; **Fig 3B**), which encodes a mannosylated cell junctional protein displayed on kidney tubular epithelia throughout the nephron [55,57–59]. By immunofluorescence microscopy of IMCD-3 cells incubated with UPEC, we observed co-localization of UPEC with Dsg2 (**Fig 3C**).

### FimH binds DSG2 *in vitro*

**Fig 4:**
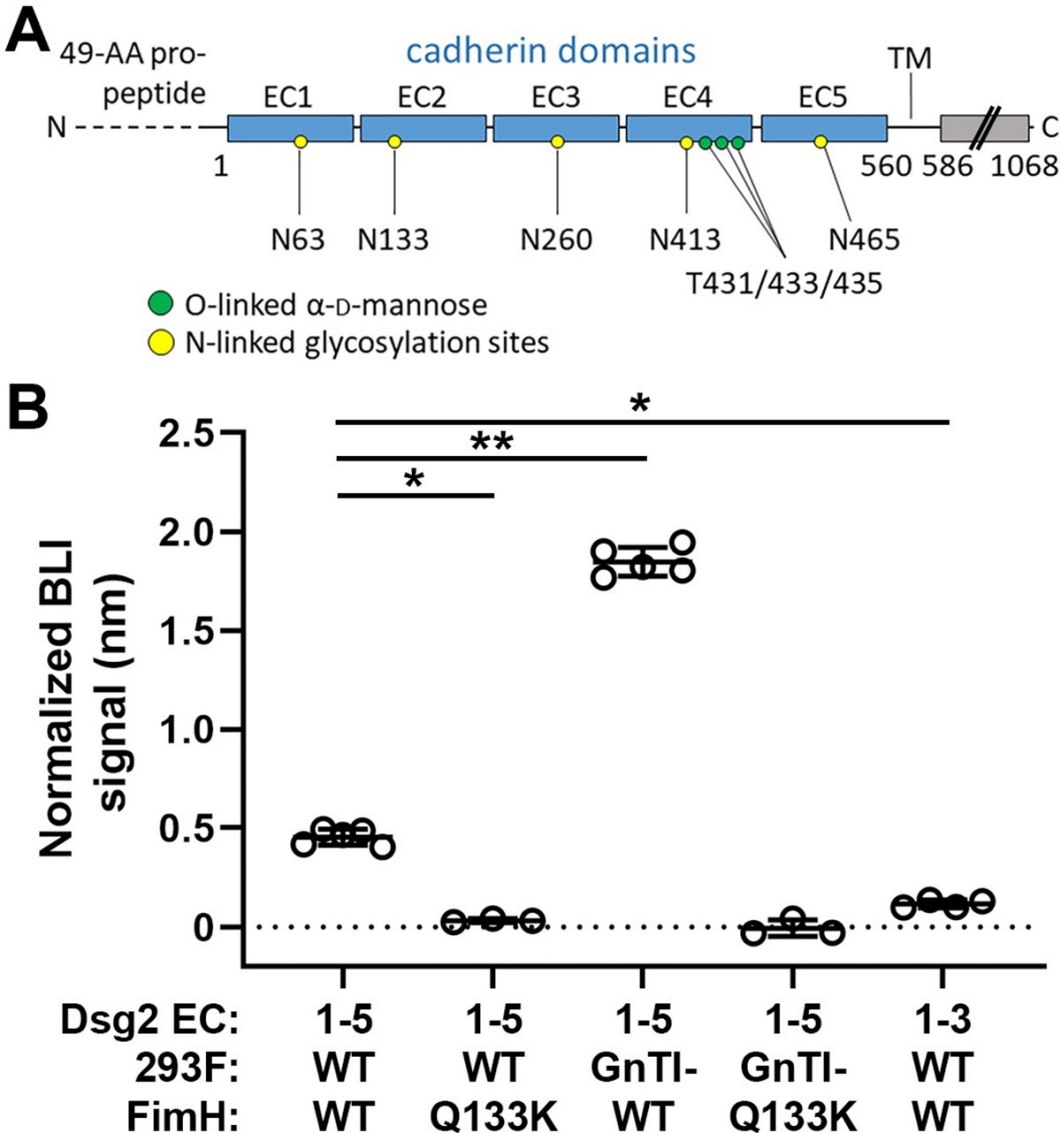
The FimH lectin domain directly binds DSG2 EC1-5 in a mannose-specific manner. **A**) Schematic of human DSG2, with five extracellular cadherin domains (EC1-5; blue) followed by a single transmembrane (TM) segment and the cytoplasmic (desmoglein) portion of the protein (gray). O-linked mannoses are represented by green dots, N-linked glycosylation sites with terminal mannose shown by yellow dots. **B**) Normalized BLI signal (mean ± SD) of DSG2 ectodomains (immobilized at 1 μg/mL) binding to FimH^LD^ or FimH^Q133K^ (10 μM). Relative BLI signals were normalized to amount of captured WT DSG2 EC1-5. DSG2 EC1-5 and DSG2 EC1-3 were purified from wild-type Expi293F cells, and DSG2 EC1-5 was also purified from Expi293F GnTI- cells (which lack complex N-glycans and instead exhibit uniform GlcNAc2Man5 glycosylation). *p = 0.036 (EC1-5 WT *vs* Q133K), **p = 0.008 (EC1-5 WT *vs* GnTI-), *p = 0.015 (EC1-5 *vs* EC1-3). n = 3–5 independent experiments per group.

Desmoglein-2 is a member of the cadherin superfamily and in humans and mice comprises five extracellular cadherin (EC) domains, a single transmembrane segment, and an intracellular domain (**Fig 4A**). To determine whether desmoglein-2 binds directly to FimH, we generated two forms of recombinant 6×His-tagged human DSG2 (EC1-5, amino acids A1-A553) [58]: in wild-type Expi293F cells, and from Expi293F GnTI- cells (which lack complex N-glycans and instead exhibit uniform GlcNAc2Man5 glycosylation). We also purified the lectin domain of FimH (FimH^LD^, amino acids F1-G160) from *E*. *coli* (**S8 Fig**). By biolayer interferometry (BLI), FimH^LD^ bound to DSG2 EC1-5 from wild-type cells with an apparent binding affinity of 1.6 μM (**Fig 4B** and **S9 Fig**). In comparison, DSG2 EC1-5 purified from GnTI- cells exhibited higher binding to FimH^LD^ (K_D,apparent_ = 484 nM, p = 0.008; **Fig 4B**), consistent with preferential binding of FimH^LD^ to highly mannosylated structures. In contrast, purified FimH^Q133K^ did not bind to either variant of DSG2 EC1-5 (p = 0.036; **Fig 4B**). Finally, FimH^LD^ bound only minimally to DSG2 EC1-3 (amino acids A1-N332; p = 0.015 *vs* EC1-5; **Fig 4B**). Taken together, these data show that FimH binds the extracellular portion of DSG2 in a mannose-dependent manner, and that DSG2 domains EC4 and/or EC5 mediate FimH binding.

**Fig 5:**
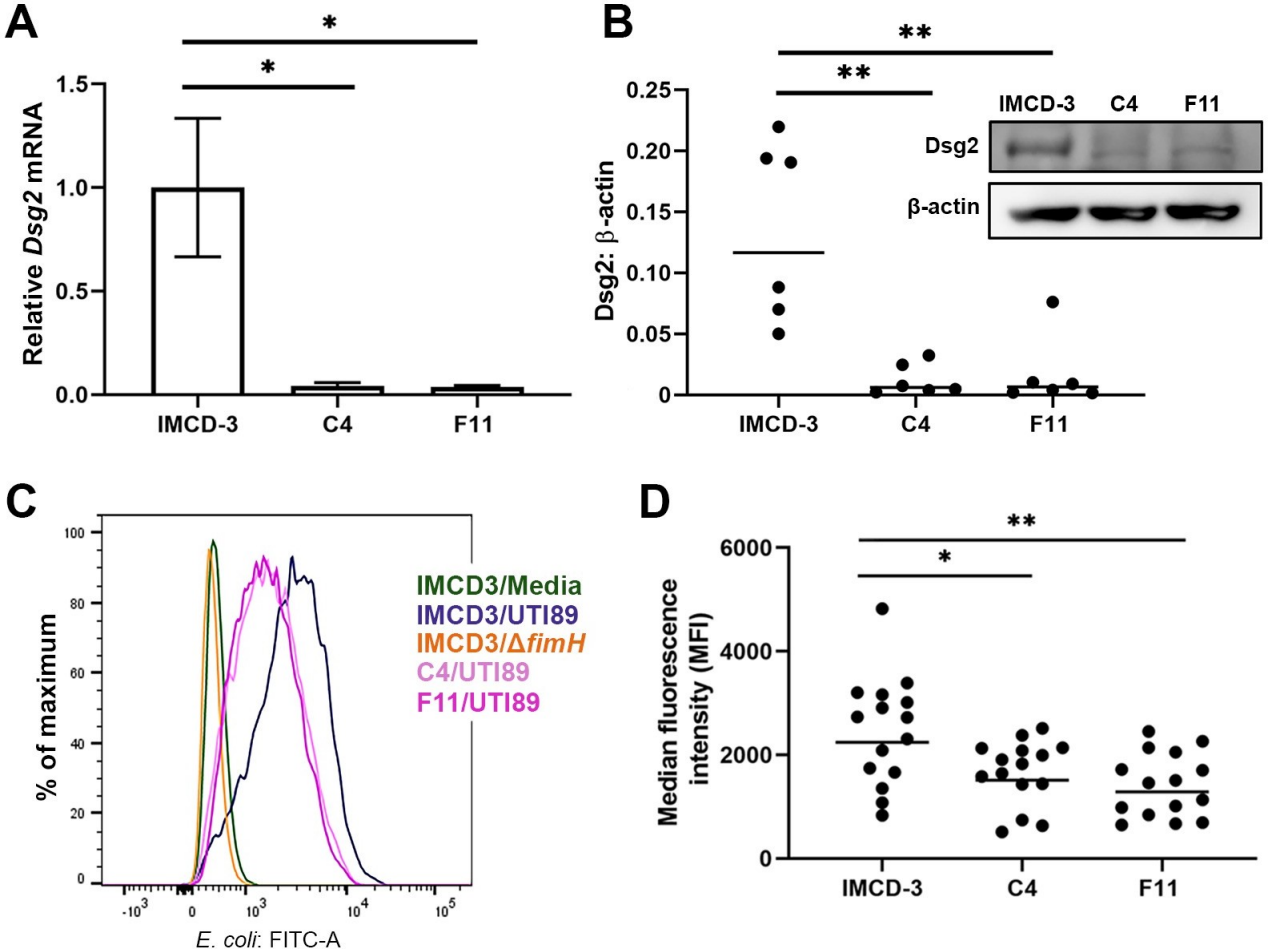
*Dsg2* knock-down cells exhibit a UPEC binding defect. **A**) qPCR of desmoglein-2 normalized to GAPDH in IMCD-3 *Dsg2* knock-down clones C4 and F11 (C4/WT *p = 0.0458, F11/WT *p = 0.0449). n = 3 independent samples per group, error bars represent standard error of the mean. **B**) Quantitative immunoblot for Dsg2 in clones C4 and F11, normalized to β-actin (median C4/WT ratio 4.3%, * p = 0.0022; median F11/WT ratio 4.7%, **p = 0.0087). Inset shows representative blot. n = 6 independent samples per group. **C, D**) Binding of UTI89 to IMCD-3 cells or to clones C4 and F11 (MOI 150) was quantified by flow cytometry after gating on single cells. **C**) Histogram of FITC shift (anti-*E*. *coli*) in infected IMCD-3 cells. Medium alone (green) or infection with Δ*fimH* reflects no shift (orange); clones C4 and F11 (light and dark pink respectively) exhibited significantly decreased UPEC binding compared to WT IMCD-3 cells (navy blue). **D**) Wild-type IMCD-3 cells infected with UTI89 displayed significantly higher median fluorescence intensity (MFI) than clones C4 and F11 (C4/WT *p = 0.0235; F11/WT **p = 0.0032). n = 15 samples per group over 5 independent experiments.

To validate Dsg2 as a UPEC receptor, we edited IMCD-3 cells with CRISPR-Cas9 to achieve specific deletion of *Dsg2*. Though hundreds of edited clones were screened, no clones entirely lacking Dsg2 expression were obtained, suggesting that complete *Dsg2* deletion is physiologically detrimental. Of note, germline *Dsg2* deletion in mice is lethal [60]. We did identify two gene-edited clones (namely C4 and F11) that exhibited sharply reduced *Dsg2* transcript expression (**Fig 5A**); by quantitative immunoblot of cell lysates, these knock-down clones expressed significantly less Dsg2 protein than wild-type IMCD-3 cells (**Fig 5B**; median C4/WT ratio 4.3%, p = 0.0022; median F11/WT ratio 4.7%, p = 0.0087). By flow cytometry, median binding of C4 and F11 cells by UPEC was decreased by 33% and 46% compared with WT IMCD-3 cells (p = 0.0235 and 0.0032, respectively; **Fig 5C and 5D**).

### Targeting the Dsg2-FimH interaction during *in vivo* infection

**Fig 6:**
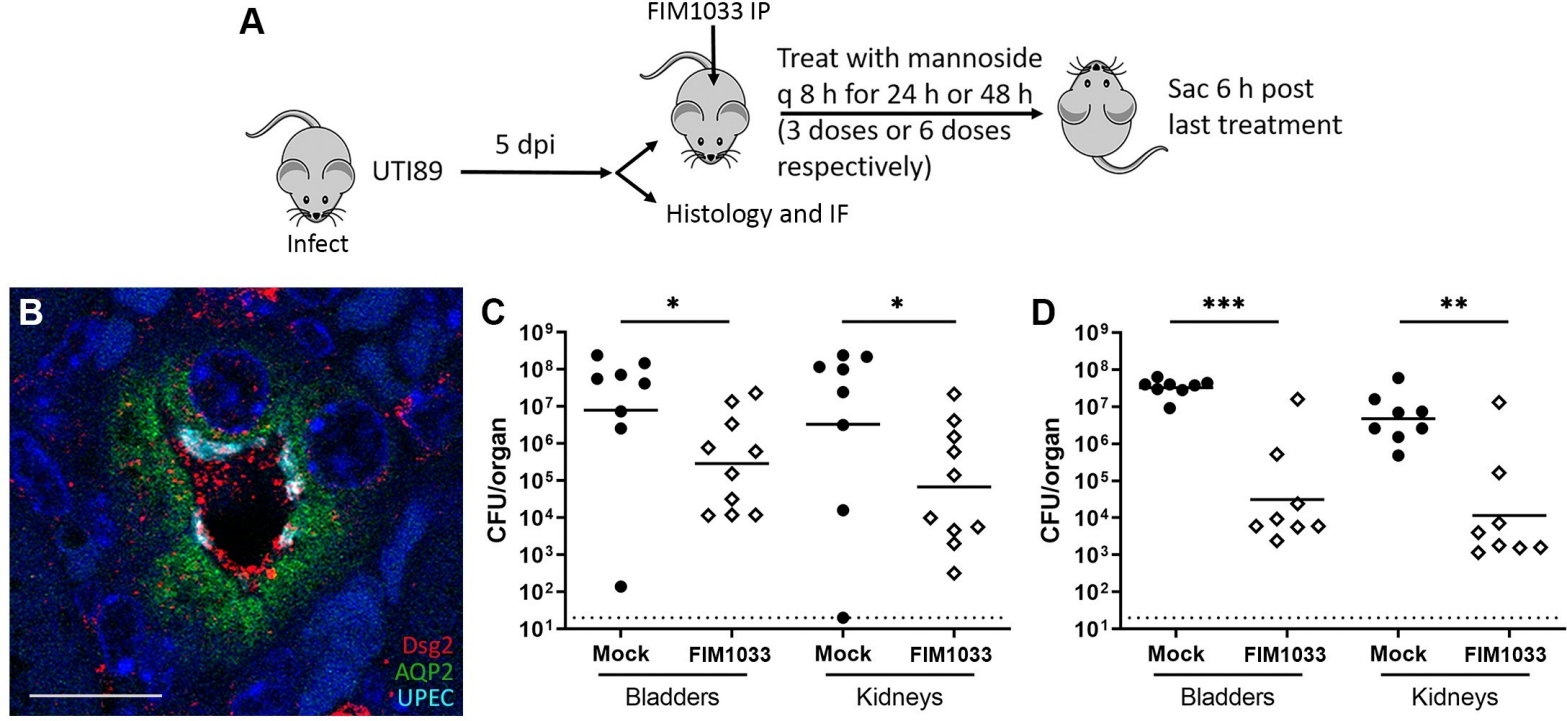
Mannoside treatment attenuates bladder and kidney infection. **A**) Schematic of mannoside treatment experiments. Male C3H/HeN mice were infected with UTI89; after 5 d, mice were administered mannoside 29R (8 mg/kg/dose intraperitoneally (IP)) or vehicle (mock treatment) every 8 h for either 24 or 48 h. **B**) Intratubular colonies of UPEC (teal) were observed 5 dpi in male C3H/HeN mice within desmoglein-2 positive (Dsg2+, red) collecting ducts (aquaporin-2 [AQP2]+, green). DAPI, blue; scale bar, 10 μm. Mannoside treatment for 24 h (**C**) significantly reduced bacterial loads in the bladder (*p = 0.0255) and kidneys (*p = 0.0434) (n = 8–10 mice per group over 2 independent experiments), while an even more significant effect was seen with 48 h treatment (**D**; bladder ***p = 0.0003, kidneys **p = 0.0042; n = 8 mice per group over 2 independent experiments).

In the kidneys of UPEC-infected C3H/HeN mice, we identified UPEC within Dsg2+, aquaporin-2+ collecting ducts 5 dpi (**Fig 6A and 6B**). Across multiple sections from several infected mice, of 239 intratubular UPEC colonies visualized 5 dpi, 228 (95%) resided in Dsg2+ tubules.

Finally, we hypothesized that if the Dsg2-FimH interaction was important for pyelonephritis, mannosides could be employed therapeutically in our mouse model. Male C3H/HeN mice were inoculated with UTI89, and early KBCs were allowed to form over 5 days [37,61]; mice were then treated with mannoside FIM1033 (formerly termed 29R [5]) (**Fig 6A**). Compared with mock-treated mice, bladder and kidney bacterial loads were significantly reduced by mannoside treatment for 24 h (kidney p = 0.0434, bladder p = 0.0255; **Fig 6C**) and even more so with treatment for 48 h (kidney p = 0.0042, bladder p = 0.0003; **Fig 6D**). These *in vivo* data support a model in which desmoglein-2 on collecting duct epithelium serves as a receptor for UPEC FimH during pyelonephritis *in vivo*.

## Discussion

In this study, we detail the importance of type 1 pili in host-pathogen interaction during ascending pyelonephritis and identify desmoglein-2 as the first receptor for FimH on renal tubular epithelium. Our findings in male C3H mice confirm that type 1 pili are essential for cystitis (as in female mice [6–11]), but more importantly provide both *in vitro* and *in vivo* evidence of the function of FimH in bacterial infection of the kidney. Furthermore, our ability to mitigate kidney infection with mannosides suggests that type 1 pilus-directed therapeutics currently under development for recurrent cystitis [2,4,5,38,39] may also be useful in pyelonephritis.

Compared to bacterial cystitis, significantly less is known about the pathogenesis of UPEC pyelonephritis, primarily due to the fact that female mice of most backgrounds resolve kidney infection spontaneously [23,24]. Work in humans has identified genetic factors that confer susceptibility to pyelonephritis and renal scarring, including polymorphisms reducing *IRF3* or *CXCR1* (encoding IL-8 receptor) expression, in certain UTI-prone kindreds [62–65]. P pili have been considered the major adhesin in kidney infection [2,19,21]. This work has been complicated by allelic variation in the P pilus adhesin PapG (UTI89 encodes the PapGIII allelic variant but does not express P pili under laboratory conditions, while the common pyelonephritis strain CFT073 expresses the PapGII variant [21]). These three alleles exhibit differing affinity for various glycolipid receptors that are differentially expressed in the kidneys of humans and model animals–including mice, where further differences exist among backgrounds [2,66]. Beyond our *in vivo* results, we found that P pili were also unnecessary for UPEC binding to IMCD-3 cells (**S6 Fig**). Finally, while P pili are enriched in the genomes of pyelonephritis-associated UPEC isolates for children with acute pyelonephritis, a significant proportion of UPEC isolates from women with acute and recurrent UTI lack P pili, suggesting a role for other adhesin(s) during ascending infection [21,67–70].

Recent works have employed new mouse models (including C3H/HeOuJ mice, male C3H/HeN mice, and androgenized female mice) which now enable detailed study of the pathogenesis of kidney infection and abscess formation [29,37,51]. In our model of ascending UTI, type 1 pili were shown to be essential for maintenance of renal infection, through both genetic mutation of the pilus and pharmacological inhibition with mannosides. Other work has hinted at a role for type 1 pili and mannose targets in kidney infection [71–75]. For instance, an early study in C3H/HeN female mice noted significant attenuation in both bladder and kidneys upon deletion of *fimH* in *E*. *coli* strain 1177 that was rescued upon complementation [75]. Further, signaling through C5a receptor 1 (C5aR1), which regulates inflammatory cell recruitment in UPEC infection, may also enhance presentation of mannosylated glycoproteins by primary renal tubular epithelial cells [72,73]. Meanwhile, experiments in which tubular infection was initiated *via* microinjection of UPEC directly into rat nephrons posited a role for type 1 pili in interbacterial interactions and biofilm formation [71]. While our work indicates that FimH mediates renal epithelial binding (in agreement with prior staining of kidney sections using purified adhesins [21,76]), the present work does not exclude a role for type 1 pili in interbacterial interactions and/or biofilm formation within the kidney. Inhibition of any of these interactions by administration of mannosides might confer therapeutic benefit in pyelonephritis.

As the C3H mouse background used here features vesicoureteral reflux, it is conceivable that ongoing bladder infection (enhanced by type 1 pili expression) may replenish the kidney niche. The converse is also likely true, as suggested by the data in **S1 Fig**, where the lack of attenuation of Δ*fimH* in the kidneys 24 hpi appears to obscure the bladder pathogenesis defect that would be expected from prior work. Thus, the infected kidneys presumably can continually re-seed the bladder niche. The absence of a kidney phenotype with Δ*fimH* 24 hpi may also indicate that an alternative host-pathogen interaction is responsible for initial UPEC binding. The timing of FimH action during UPEC kidney colonization is therefore an exciting avenue for future study.

Using a CRISPR-Cas9 screen, we identified desmoglein-2 as a receptor for UPEC during ascending infection. DSG2 is a member of the cadherin family of Ca^2+^-binding proteins, involved in intercellular junctions *via* the desmosome [77,78]. Its identified roles in mammalian disease states are limited; of note, cardiomyocyte-specific conditional knockout of *Dsg2* in mice phenocopies human arrhythmogenic cardiomyopathy, which is correlated with loss-of-function *DSG2* mutations [79,80]. Interestingly, other members of the desmoglein family also act as receptors for unrelated microbes [81]. Dsg2 has been specifically implicated as a receptor for group B adenovirus serotypes 3, 7, 11, and 14 [82,83]; of note, serotype 11 is most commonly associated with hemorrhagic cystitis in renal and other transplant recipients [84]. Along the length of the nephron, DSG2 expression is highest within the collecting duct and decreases as one ascends to more proximal segments of the tubule [59]. As a family, desmogleins (and other cadherins) exhibit a unique form of mannosylation in which α-d-mannose (the binding target of type 1 pili) is present as a novel O-linked glycosylation modification [58]. Prior studies have demonstrated FimH binding to N-linked glycans containing a terminal mannose [7,10,85,86]. While N-linked glycans are present on each EC domain of DSG2 [58], our data suggest that FimH interaction with native DSG2 is mediated through EC4 and/or EC5 (**Fig 4**). Of particular interest is the possibility that the cadherin-specific O-linked α-d-mannose on EC4 may represent the preferred binding target. While FimH canonically binds mannose, existing studies have not aimed to interrogate whether the spatial arrangement of nearby amino acid residues may also influence binding affinity. Future work, including determination of the structural basis of DSG2-FimH binding, will further address this question.

One limitation of this study is that we were unable to obtain a complete *Dsg2* knockout cell line, indicating that complete absence of Dsg2 may confer a significant defect in *in vitro* growth of IMCD-3 cells. It is becoming better appreciated that CRISPR-generated knockout cell clones display phenotypic plasticity, and residual low-level protein expression often persists [87]. Therefore, it is difficult to precisely quantify what proportion of UPEC binding is to Dsg2 as opposed to other mannosylated cell-surface receptors. It is possible that Dsg2 knock-down leads to compensatory production of other proteins; that Dsg2 knock-down reveals the participation of more minor binding partners for type 1 pili; or that the amount of residual Dsg2 in the knock-down clones provides sufficient receptors for UPEC to bind a modest proportion of cells. The present data do not distinguish among these possibilities.

Our results indicate that Dsg2 may not be the sole receptor for UPEC within the kidney, but rather the most important receptor among others on renal tubular epithelium. The apical surfaces of bladder epithelial facet cells are coated with a comparatively restricted set of proteins, largely four uroplakins [88]. Among these, uroplakin Ia bearing N-linked oligomannose is thought to be the primary receptor important for UPEC binding of intact bladder epithelium [6–8,10,11,89]. In contrast, the renal tubular epithelium bears a wider variety of surface proteins, a number of which might be mannosylated. Of note, CRISPR knock-down of the glycosyltransferase Dad1 on IMCD-3 cells also conferred a UPEC binding defect (**Fig 3B**), suggesting that this enzyme may play a role in mannosylation of Dsg2 and/or other potential receptors for UPEC type 1 pili. Additionally, multiple innate immune genes were statistically enriched in the CRISPR screen (**Fig 3B**), hinting that innate response pathways may alter receptor expression and thereby influence UPEC binding; this too represents an avenue for future study.

The present work highlights the importance of type 1 pili and the mannosylated epithelial receptor desmoglein-2 in UPEC colonization of renal tubules. As the type 1 pilus adhesin FimH is already targetable by small-molecule inhibitors and vaccines in patients with cystitis [3,68,90], these therapeutics may prove useful in prevention or treatment of upper-tract UTI as well. Finally, desmoglein-2 is expressed widely across many epithelia [55,57,91,92], suggesting that it may serve as a FimH receptor in other UTI-relevant niches, perhaps as a secondary receptor in the urinary bladder or in the gut.

## Materials and methods

### Ethics statement

All animal protocols complied with relevant ethical regulations and received prior approval from the Institutional Animal Care and Use Committee at Washington University (approval number 20180159).

### Bacterial strains and growth

For mouse infections and for binding studies with IMCD-3 cells, bacteria were grown statically at 37°C for 18 h in Luria-Bertani (LB) broth. For the CRISPR screen, type 1 pili were induced by static growth at 37°C for 18 h, then 1:100 subculture and static growth for an additional 18 h. Bacterial strains were all previously published, with mutations generated using the λ Red Recombinase system [93] (see **S2 Table**). Bacteria were pelleted at 7,500 × *g* at 4°C for 10 min, then resuspended to an OD_600_ of 1.0 (~4 × 10^8^ colony-forming units [CFU]/mL) in sterile phosphate-buffered saline (PBS) for mouse infections, or in DMEM F-12 (Invitrogen 1132–0033) supplemented with 10% fetal bovine serum (FBS; VWR 97068–085) for tissue culture experiments.

### Mouse infections

Male C3H/HeN mice (Envigo) aged 8–9 weeks were infected as previously described [29]. Briefly, mice were anesthetized with inhaled 3% isoflurane, and the lower abdomen was shaved and sterilized with 2% chlorhexidine solution. A 3-mm midline abdominal incision was made through the skin and peritoneum, exposing the bladder. The bladder was aseptically emptied before 50 μl of inoculum (1–2 × 10^7^ CFU in PBS) was injected into the bladder lumen *via* 30-gauge needle over 10 s. The bladder was allowed to expand for an additional 10 s before the needle was removed. The peritoneum and skin incisions were closed with simple, interrupted sutures. At the time of surgery, mice were given sustained-release buprenorphine (1 mg/kg subcutaneously) for analgesia. At the indicated time points, mice were euthanized by CO_2_ asphyxiation. Bladders and kidney pairs were sterilely removed and homogenized in 1 mL or 0.8 mL PBS, respectively. Homogenates were serially diluted and plated on LB agar for CFU enumeration.

### Immunofluorescence microscopy

Infected bladders and kidneys were bisected and fixed in 10% neutral buffered formalin for 24 h. Fixed tissues were embedded in paraffin, sectioned, and stained. Unstained slides were deparaffinized in xylenes, rehydrated in isopropanol, boiled in 10 mM sodium citrate, and blocked in 1% bovine serum albumin (BSA), 0.3% Triton-X 100 in PBS for 1–2 h at room temperature (RT). Tissue culture cells were fixed in methanol at -20°C for 10 min and then blocked in 3% BSA for 1 h. Slides were incubated with primary antibodies for 4 h or overnight at RT and then (after washing in PBS) with secondary antibodies for 1 h at RT. Slides were then mounted with ProLong Gold antifade reagent (Invitrogen) and images acquired on an Olympus FV1200 confocal microscope. Primary antibodies utilized were: rabbit anti-type 1 pili (1:500 dilution), rabbit anti-*E*. *coli* (1:1000 dilution, E3500-06C; US Biological), goat anti-*E*. *coli* (1:100–1:500 dilution, B65109G; Meridian Life Science), mouse anti-AQP2 conjugated with AlexaFluor 488 or 647 (1:50–1:200 dilution, sc-515770; Santa Cruz Biotechnology), mouse anti-desmoglein 2 (1:100 dilution, 6D8, MCA2272T, BioRad), rabbit anti-desmoglein 2 (1:100, A303-758A, Bethyl Labs). Secondary antibodies utilized were AlexaFluor 488-conjugated goat anti-rabbit IgG (1:500 dilution, A11008; Life Technologies), AlexaFluor 647-conjugated chicken anti-rabbit IgG (1:200, A-21443;ThermoFisher), AlexaFluor 594-conjugated donkey anti-goat IgG (1:200, ab150132; abcam), Cy5-conjugated donkey anti-mouse IgG (1:250, 715-175-151; JacksonImmuno), AlexaFluor 488-conjugated goat anti-rabbit IgG (1:1000, 111-095-144; JacksonImmuno). Nuclear staining was with DAPI (1:10,000) or SYTO 61 (1:1000, Molecular Probes).

### Tissue culture

Murine intramedullary collecting duct cells (IMCD-3; ATCC CRL-2123) were cultured in DMEM F-12 supplemented with 10% FBS. Medium included 0.1 mg/mL penicillin-streptomycin (Gibco 15140–122) until the day before an experiment, when medium was changed to the above without antibiotics. Cells were maintained at 37°C in a humidified atmosphere with 5% CO_2_.

### *In vitro* binding assays

The day before infection, IMCD-3 cells were seeded in 24-well plates. Medium was removed and cells were washed with PBS supplemented with Mg^2+^ and Ca^2+^ (PBS-MgCa; Sigma-Aldrich D8662). After inoculation with UPEC at a multiplicity of infection (MOI) of 20, plates were centrifuged at 400 × *g* for 3 min, then returned to the incubator. To enumerate bound CFU, after 45 min, wells were washed 5 times with PBS-MgCa, then lysed with 0.1% Triton X-100 (Sigma T9284). To enumerate internalized CFU, after 60 min, cells were washed with PBS-MgCa, treated with medium containing 50 μg/mL gentamicin (Thermo Fisher 15750060), and incubated at 37°C for 90 min, then lysed with 0.1% Triton X-100. Lysates were serially diluted and plated to LB agar. Binding efficiencies were calculated in comparison to input wells, in which cells were inoculated with UPEC, incubated for 45 min, then lysed by addition of Triton X-100, and the well contents plated to LB agar for enumeration of input CFU.

### Flow cytometry

The day before infection, IMCD-3 cells were seeded in 6-well plates. Cells were infected with UPEC (at MOI 20–150) for 45 min, then washed as described above; cells were liberated with 0.05% trypsin-0.02% EDTA (Gibco), pelleted, and fixed in 4% paraformaldehyde (Electron Microscopy Sciences) for 30–60 min at RT. Cells were then blocked in 3% BSA for 1 h at RT. Cells were stained with primary rabbit anti-*E*. *coli* antibody (1:500 dilution, E3500-06C; US Biological) or isotype control (Rabbit serum; Sigma-Aldrich R9133) and secondary AlexaFluor 488-conjugated donkey anti-rabbit IgG (Invitrogen A21206) for 1 h each. Samples were stored in 3% BSA until analyzed on a Becton Dickinson (BD) LSR II Fortessa flow cytometer. Gating strategy is shown in **S10 Fig.**

### Generation of virus and viral constructs

Viruses were generated by transfecting 293T cells with lentiviral cDNA (VSV-G, pMD2.G, Addgene #12259; psPAX2, Addgene #12260; and construct of interest) using Opti-MEM (Thermo Fisher 31985062) and TransIT LT1 transfection reagent (Mirus MIR2300). Virus was harvested 48 h post transfection.

### Library preparation and CRISPR screen

IMCD-3 cells were transduced with lentivirus containing Cas9 (pXPR_101, Addgene #52962) by spinoculation (1 mL of Cas9 lentivirus onto 3 × 10^6^ cells), then selected with 5 μg/mL blasticidin (ThermoFisher A1113903). Cas9 activity was assayed by transducing cells with lentivirus containing pXPR_011 (Addgene #59702), which expresses eGFP and a sgRNA targeting eGFP, and selecting with 5 μg/mL puromycin (ThermoFisher A1113803). Loss of GFP expression in cells expressing the eGFP guide was assessed by flow cytometry as described above. After Cas9 activity was confirmed, a Brie mouse sgRNA library (Brie pXPR_003, Batch 3, Lot #m-AA89-20171107; generated by the Broad Institute) was transduced at MOI 0.5 into IMCD-3 Cas9 cells by spinoculation and selected with 5 μg/mL puromycin for 10 d until screening.

For screening, 5 × 10^6^ IMCD-3 cells were seeded into eighty 15-cm dishes the day before infection. Cells were infected with UTI89 at MOI 150 and incubated at 37°C for 45–75 min, then fixed and stained for flow cytometry as described above. Samples were placed at 4°C overnight on a tube roller; the following day, the 5% least FITC-positive cells were collected on a Sony iCyt Synergy BSC sorter. Tubes containing these low-FITC sorted cells were centrifuged, resuspended in 250 μl PBS, and stored at -20°C. A total of 6 × 10^8^ mock-treated cells were harvested, separated into aliquots of 8 × 10^7^ cells, resuspended in 2 mL PBS, and stored at -20°C until genomic DNA preparation. DNA was extracted using QIAamp DNA Blood Maxi kit (Qiagen 51192) for mock samples and QIAamp DNA FFPE Tissue kit (Qiagen 56404) for experimental samples.

### Sequencing and bioinformatics

Illumina sequencing was performed as described previously [53]. Briefly, genomic DNA was aliquoted into multiple wells of a 96-well plate (up to 10 μg of DNA in 50 μL total volume). Samples were sequenced on an Illumina HiSeq 2000. Barcodes in the P7 primer were deconvoluted, and the sgRNA sequence was mapped to a reference file of sgRNAs in the Brie library. To normalize for different numbers of reads per condition, read counts per sgRNA were normalized to 10^7^ total reads per sample; this normalized value was then log_2_ transformed. We used the hypergeometric distribution method to rank sgRNAs and calculate gene p-values using the probability mass function of a hypergeometric distribution (https://portals.broadinstitute.org/gpp/public/analysis-tools/crispr-gene-scoring-help). We considered candidate genes those having an average log fold change >0.5 and a false discovery rate >2.5 [53,54]. For input of the hypergeometric distribution ranking, we subtracted the log_2_ normalized read values of the uninfected unsorted IMCD-3 Brie library from the log_2_ normalized read values of the 5% lowest UPEC-bound sorted cells. We used R Studio to visualize the results of the hypergeometric distribution analysis.

### Generation of *Dsg2* knock-down clones (C4 and F11)

*Dsg2* knock-down clones C4 and F11 were among hundreds generated by the Genome Engineering and iPSC Center at Washington University School of Medicine. IMCD-3 cells were nucleofected with Cas9 and a Dsg2-specific sgRNA (5’ GGAACTACGCATCAAAGTTCTGG 3’). Single-cell clones were isolated by FACS and expanded in 96-well plates. Cells were harvested, and genomic DNA was amplified and subjected to targeted deep sequencing of a ~400-bp amplicon flanking the gRNA target. Clones were screened for frameshifts by sequencing the target region with Illumina MiSeq at ~1500× coverage. We obtained no clones that completely lacked *Dsg2* expression. Frameshift mutations identified in knock-down clones C4 and F11 are detailed in **S3 Table**.

### Quantitative PCR

RNA was isolated from tissue culture cells using the Qiagen RNeasy kit. qPCR was performed using the Applied Biosystems TaqMan RNA-to-Ct 1-Step Kit (ThermoFisher 4392938) and the probes listed in **S4 Table** (Integrated DNA Technologies).

### Immunoblot

Samples were separated by SDS-PAGE and transferred to a polyvinylidene difluoride (PVDF) membrane (Millipore) using overnight wet transfer (48mM Tris pH 9.2, 38mM glycine, 20% methanol, 0.05% SDS). After blocking for 6–8 h with 5% milk in PBS-T, blots were probed with rabbit anti-desmoglein-2 (1:250 dilution, A303-758A; Bethyl Labs) and mouse anti-β-actin (1:100,000 dilution, 3700; Cell Signaling) in PBS-T with 5% milk overnight. Blots were then secondarily probed with HRP-conjugated sheep anti-mouse IgG (1:1000 dilution, NA931V; GE) and HRP-conjugated donkey anti-rabbit IgG (1:1000 dilution, NA934V; GE) for 1 h. Gels were developed using Clarity and Clarity Max Western ECL substrates (1705060 and 1705062; BioRad). Western blot images were analyzed with ImageJ.

### Protein production and biolayer interferometry (BLI)

Methods for purification of full-length FimH and the lectin domain (FimH^LD^; amino acids 1–160) have been described previously [10]. Briefly, untagged FimH^LD^ was purified from *E*. *coli* periplasmic extracts using ion-exchange and size-exclusion chromatography, dialyzed into sterile PBS, and stored at 4°C until use.

Expression constructs for 6×His-tagged human DSG2 EC1-5 (amino acids A1-A553) or DSG2 EC1-3 (amino acids A1-N332) (kind gifts of O. Harrison and L. Shapiro [58]) were transfected (1 μg/mL of culture) into Expi293F cells (ThermoFisher) using 293fectin and Opti-MEM. Six days post transfection, the supernatant containing the 6×His-tagged DSG2 ectodomains was collected, dialyzed into 20mM Tris, 150mM NaCl pH 8 overnight at 4°C, batch purified by nickel-nitrilotriacetic acid (Ni-NTA) affinity chromatography, and then further purified by HiLoad 16/600 Superdex 200 size-exclusion chromatography (GE Healthcare). Protein was concentrated and was exchanged into a final buffer of sterile PBS, then stored at 4°C until use in binding experiments. DSG2 EC1-5 was also expressed in Expi293F GnTI- cells (which generate proteins lacking complex N-linked glycans but decorated only with high mannose) and purified as described above.

All BLI experiments were performed in 10 mM HEPES (pH 7.4), 150 mM NaCl, 1 mM CaCl_2_, 3 mM EDTA, and 0.005% P20 surfactant with 1% BSA at 25°C using an Octet-Red96 device (Pall ForteBio). DSG2 variants were biotinylated using EZ-Link NHS-PEG_4_-Biotin (Thermo Fisher) per manufacturer’s instructions. Biotinylated DSG2 was loaded onto streptavidin biosensors (ForteBio), then incubated with different concentrations of FimH^LD^ or FimH^Q133K^ (10 μM to 156 nM) for 15 min followed by a 20 min dissociation. Real-time data were analyzed using BIAevaluation 3.1 (GE Healthcare), and kinetic curves were fitted using a global 1:1 binding algorithm with drifting baseline.

### Mannoside treatment

Male C3H/HeN mice were infected as described above. Beginning 5 dpi, mice were given mannoside FIM1033 (gift of Fimbrion Therapeutics), 8 mg/kg in sterile PBS with 4% DMSO, by intraperitoneal injection every 8 h for 24 or 48 h. Mock-treated mice were injected with 4% DMSO in sterile PBS. Mice were sacrificed 6 h post last treatment dose.

### Statistical analysis

Statistical analysis was performed using Prism 8 (GraphPad Software). Differences were analyzed with the unpaired, two-tailed, nonparametric Mann-Whitney U test. P values <0.05 were deemed significant.

## Supporting information

S1 Fig Male C3H/HeN mice were infected with UTI89 (closed circles) or UTI89Δ*fimH* (open diamonds), and organs were harvested 24 hpi. No significant differences in bacterial loads between WT and Δ*fimH* were observed, indicating that Δ*fimH* reaches the kidney normally after inoculation of the bladder. Horizontal bars indicate geometric mean, and dotted line indicates limit of detection. n = 9 mice per condition over 2 independent experiments.
(TIF)

S2 Fig Male C3H/HeN mice were infected with CFT073 (closed circles) or CFT073Δ*fimH* (open diamonds), and organs were harvested (A) 24 hpi or (B) 2 wpi. No significant differences in bacterial loads were observed 24 hpi; Δ*fimH* was attenuated significantly 2 wpi in both the bladder (*p = 0.0482) and kidneys (****p<0.0001). Horizontal bars indicate geometric mean, and dotted line indicates limit of detection. **A**) n = 10 mice per condition over 2 independent experiments; **B**) n = 13–14 mice per condition over 3 independent experiments.
(TIF)

S3 Fig Male C3H/HeN mice were infected with UTI89 (closed circles) or isogenic, individual UTI89 CUP pili mutants (open diamonds), and organs were harvested 2 wpi. None of these CUP pili mutants displayed defects in bladder or kidney colonization. Horizontal bars indicate geometric mean, and dotted line indicates limit of detection. n = 6–18 mice per experimental condition over 6 independent experiments.
(TIF)

S4 Fig Male C3H/HeN mice were co-infected with antibiotic-tagged UTI89 (closed circles) and each UTI89 CUP pilus mutant (open triangles). Bladders (**A**) and kidneys (**B**) were harvested 2 wpi and homogenates plated on selective media. Other than Δ*fimH*, none of the CUP pilus mutants displayed defects in bladder or kidney colonization. Horizontal bars indicate geometric mean, and dotted line indicates limit of detection. n = 3–9 mice per condition over 5 independent experiments.
(TIF)

S5 Fig Functional variants of type 1 pili are ineffective in establishing kidney infection. Male C3H/HeN mice were infected with UTI89 (closed circles) or with the indicated type 1 pili variants. Bladders and kidneys harvested 2 wpi yielded higher bacterial loads of wild-type (WT) UTI89 compared to (A) UTI89 FimH_A27V/V163A_ (open diamonds) (bladder ****p<0.0001, kidney ***p = 0.0001) or (B) UTI89 FimA_A22R_ (open diamonds) (bladder ****p<0.0001, kidney ***p = 0.0038). Horizontal bars indicate geometric mean, and dotted line indicates limit of detection. A) n = 13–19 mice per condition over 3 independent experiments; B) n = 10–15 mice per condition over 2 independent experiments.
(TIF)

S6 Fig Type 1 pili mediate UPEC binding to IMCD-3 cells. Cultured murine collecting duct cells were infected with the indicated UPEC strains, and cells were stained with anti-*E*. *coli* antibody and analyzed by flow cytometry; median fluorescence intensities are shown. Mutation of *fimH* in UTI89 abrogated binding, while deletion of the P pilus usher (*papC*) had no effect. Chromosomal re-integration of wild-type *fimH*, but not *fimH*^Q133K^, restored binding in the UTI89 *fimH* mutant. Deletion of *fimH* in CFT073 similarly abrogated binding by this urosepsis strain. n = 9–10 wells per condition (aggregate of three triplicate experiments). ****p<0.0001.
(TIF)

S7 Fig Methyl α-d-mannopyranoside and mannoside FIM1033 inhibit UPEC binding to collecting duct epithelial cells. **A**) FITC signal (anti-*E*. *coli*) on IMCD-3 cells infected with wild-type UTI89 (navy blue) compared to infection with UTI89 Δ*fimH* (orange) or with wild-type UTI89 in media containing 2% methyl α-d-mannopyranoside (teal). **B**) Binding of IMCD-3 cells by UTI89 (closed circles) with or without addition of methyl α-d-mannopyranoside, or with Δ*fimH* (open diamonds), was quantified by flow cytometry after gating on single cells. Significance is shown in comparison to WT UTI89 without methyl α-d-mannopyranoside (**p<0.01, ***p<0.001). n = 6–9 samples per condition over 3 independent experiments. **C**) FITC signal (anti-*E*. *coli*) on IMCD-3 cells infected with wild-type UTI89 (navy blue) compared to infection with UTI89 Δ*fimH* (orange) or with wild-type UTI89 in media containing FIM1033 (0.1μM, light pink; 1μM, dark pink; 10μM, maroon). **D**) Binding of IMCD-3 cells by WT UTI89 (closed circles) with or without addition of FIM1033, or by Δ*fimH* (open diamonds), was quantified by flow cytometry after gating on single cells. Horizontal bars indicate geometric mean, and significance is shown in comparison to WT UTI89 without FIM1033 (****p<0.0001). n = 9 samples per condition over 3 independent experiments.
(TIF)

S8 Fig Purification of 6×His-tagged DSG2 EC1-5 from Expi293F cells. **A**) Coomassie blue-stained gel of FimH_LD_ and FimH_Q133K_. **B**) Coomassie blue-stained gel after metal-affinity purification of DSG2 EC1-5 and EC1-3 from cell supernatant. DSG2 EC1-5 was expressed in both WT Expi293F cells as well as in Expi293F GnTI- cells (lacking complex glycans). In right panel, due to its immunoglobulin folds, addition of the reducing agent DTT alters the apparent molecular weight (MW) of DSG2 EC1-5, from ~70 kDa to ~75 kDa.
(TIF)

S9 Fig Biolayer interferometry (BLI) tracings for binding of FimH^LD^ to immobilized DSG2 EC1-5 expressed from WT Expi293F cells (left panel) or Expi293F GnTI- cells (right panel). A 1:1 binding model (red lines) was used to fit experimental curves (black lines). Representative curves shown from 5 independent BLI runs per condition; affinity values represent the mean ± SD of two independent experiments.
(TIF)

S10 Fig Gating strategy for flow cytometry experiments. Samples were gated on single cells. Representative samples of IMCD-3 cells treated with medium alone (red) and WT UTI89 (blue) shown.
(TIF)

S1 Table CRISPR screen analyzed ranks and statistics. (XLSX)

S2 Table Strains used in this study. (DOCX)

S3 Table Frameshift mutations within *Dsg2* in IMCD-3 clones C4 and F11 (two allelic variants present in each clone are shown). (DOCX)

S4 Table Probes and primers for qPCR. (DOCX)
