# Supplementary figures and images for: A host receptor enables type 1 pilus-mediated pathogenesis of Escherichia coli pyelonephritis
Source: PLoS Pathog. 2021 Jan 29;17(1):e1009314. doi: 10.1371/journal.ppat.1009314 (PMC7875428; doi:10.1371/journal.ppat.1009314)

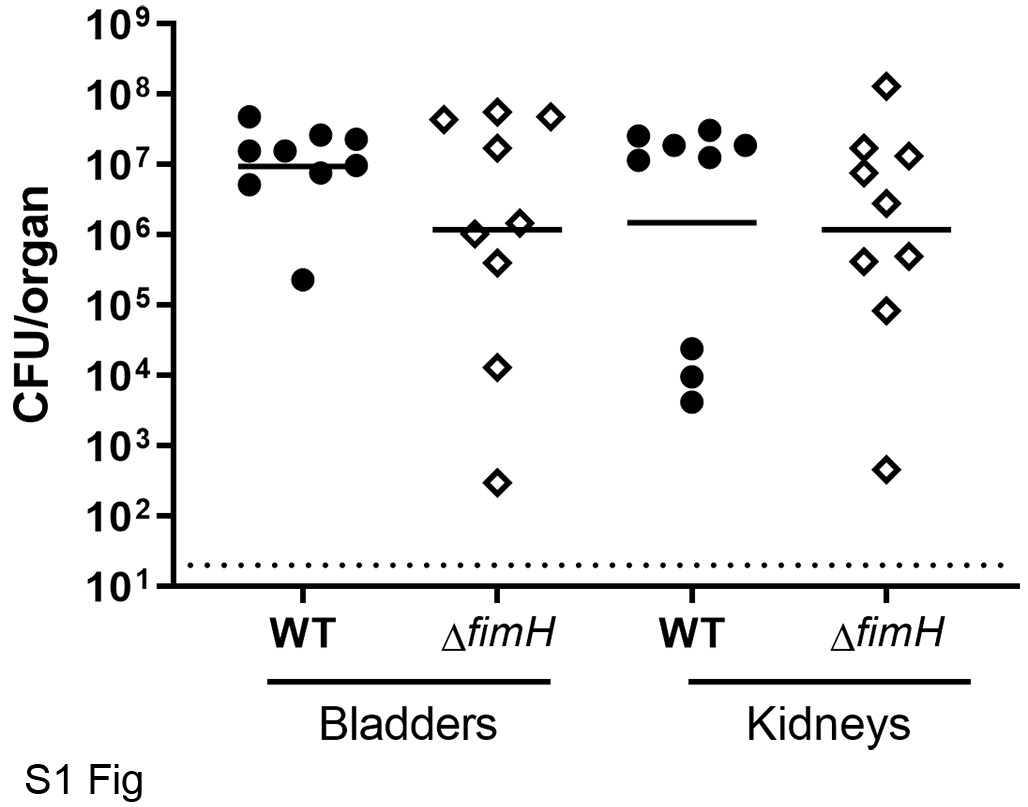

Supplement: S1 Fig — No significant differences in bacterial loads between WT and ΔfimH were observed, indicating that ΔfimH reaches the kidney normally after inoculation of the bladder. Horizontal bars indicate geometric mean, and dotted line indicates limit of detection. n = 9 mice per condition over 2 independent experiments. (TIF) [file ppat.1009314.s001.tif]

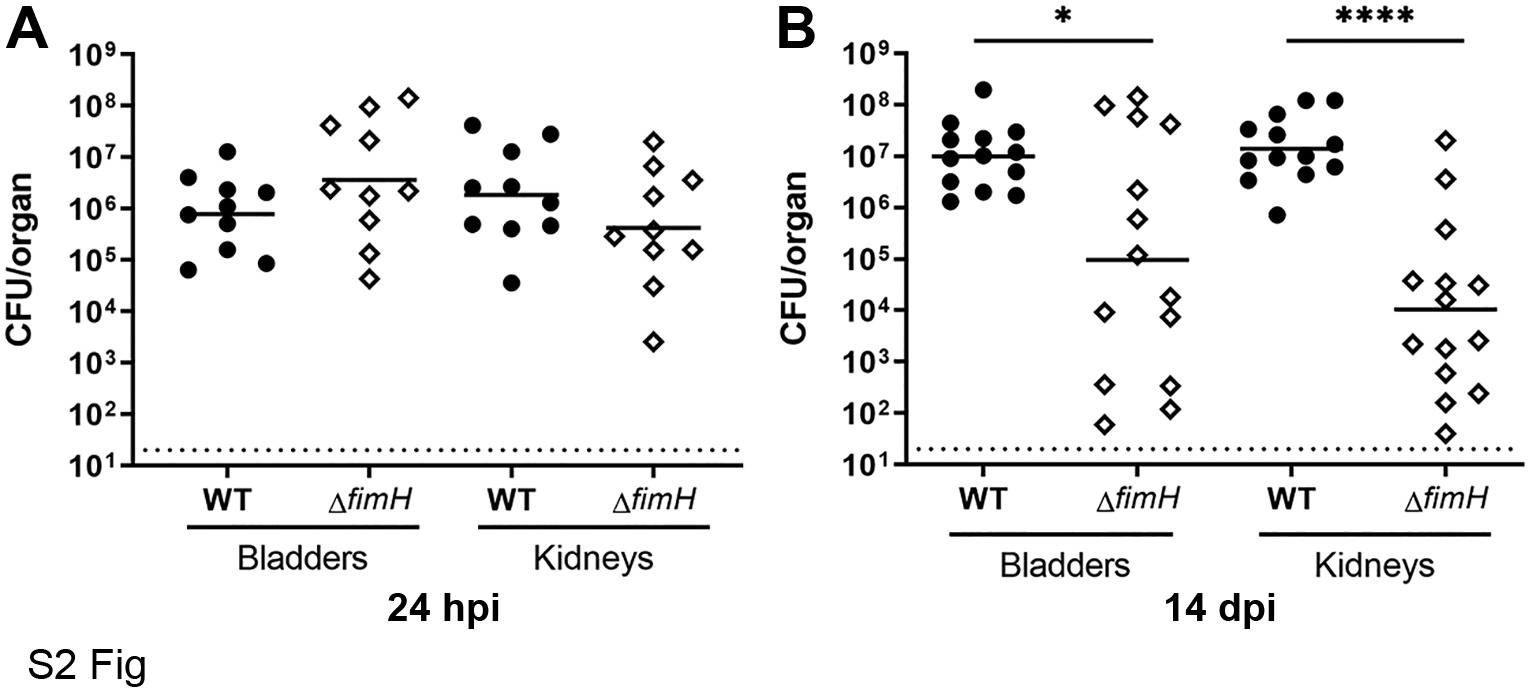

Supplement: S2 Fig — No significant differences in bacterial loads were observed 24 hpi; ΔfimH was attenuated significantly 2 wpi in both the bladder (*p = 0.0482) and kidneys (****p<0.0001). Horizontal bars indicate geometric mean, and dotted line indicates limit of detection. A) n = 10 mice per condition over 2 independent experiments; B) n = 13–14 mice per condition over 3 independent experiments. (TIF) [file ppat.1009314.s002.tif]

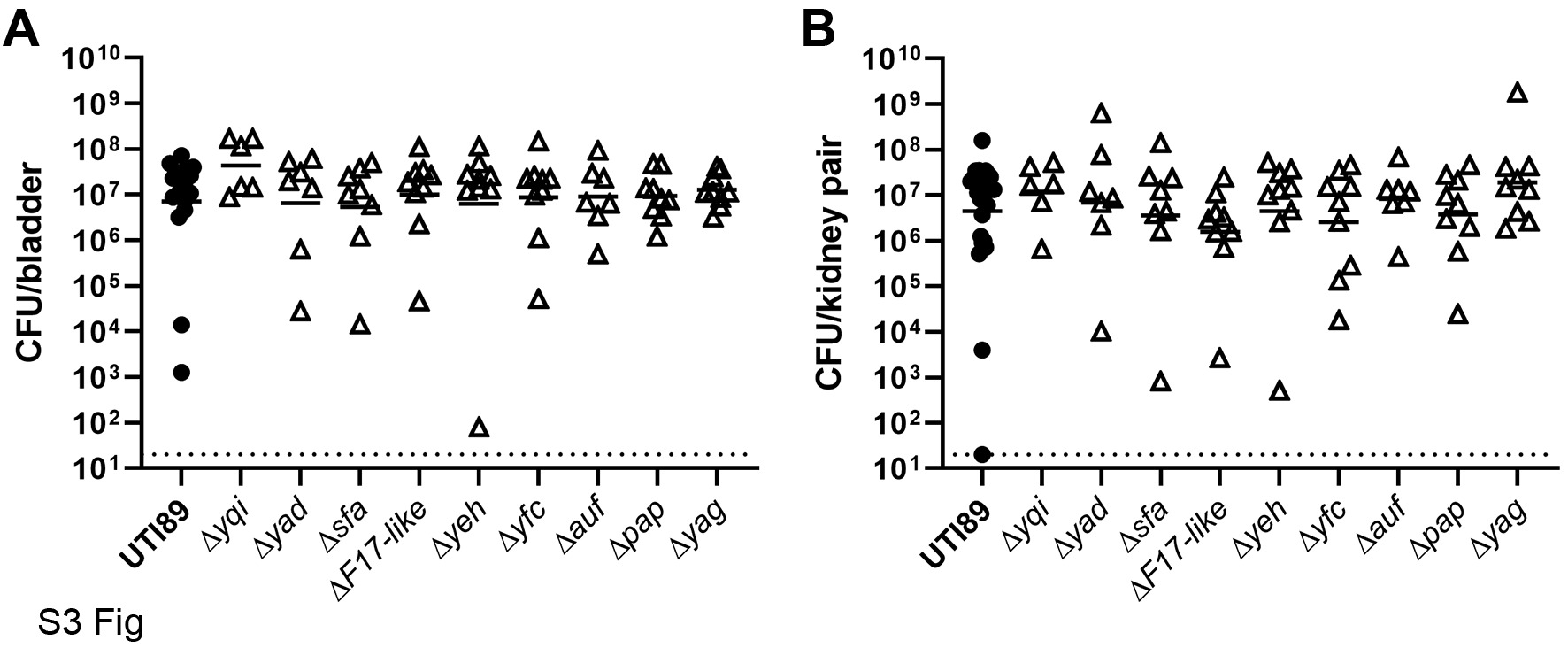

Supplement: S3 Fig — None of these CUP pili mutants displayed defects in bladder or kidney colonization. Horizontal bars indicate geometric mean, and dotted line indicates limit of detection. n = 6–18 mice per experimental condition over 6 independent experiments. (TIF) [file ppat.1009314.s003.tif]

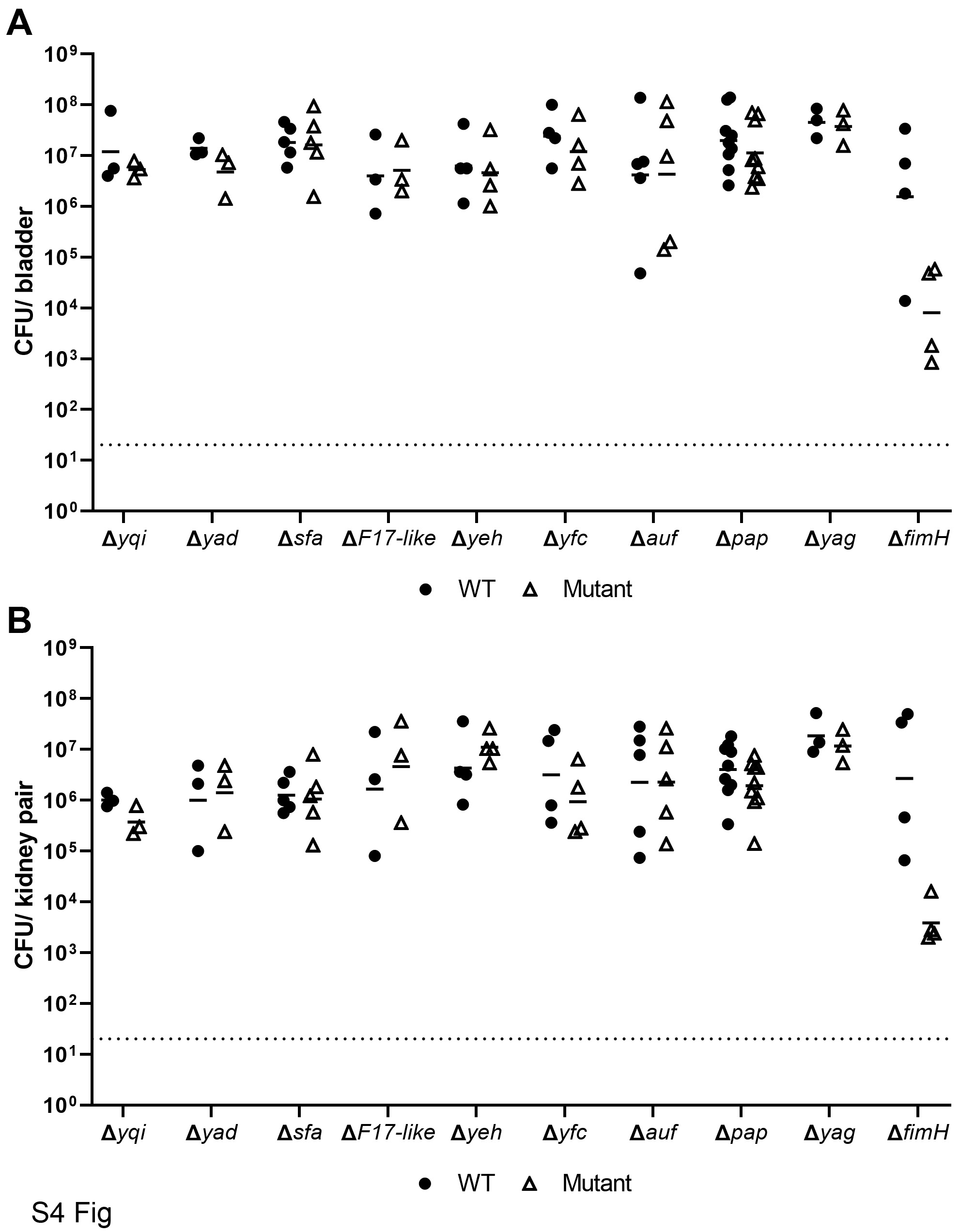

Supplement: S4 Fig — Bladders (A) and kidneys (B) were harvested 2 wpi and homogenates plated on selective media. Other than ΔfimH, none of the CUP pilus mutants displayed defects in bladder or kidney colonization. Horizontal bars indicate geometric mean, and dotted line indicates limit of detection. n = 3–9 mice per condition over 5 independent experiments. (TIF) [file ppat.1009314.s004.tif]

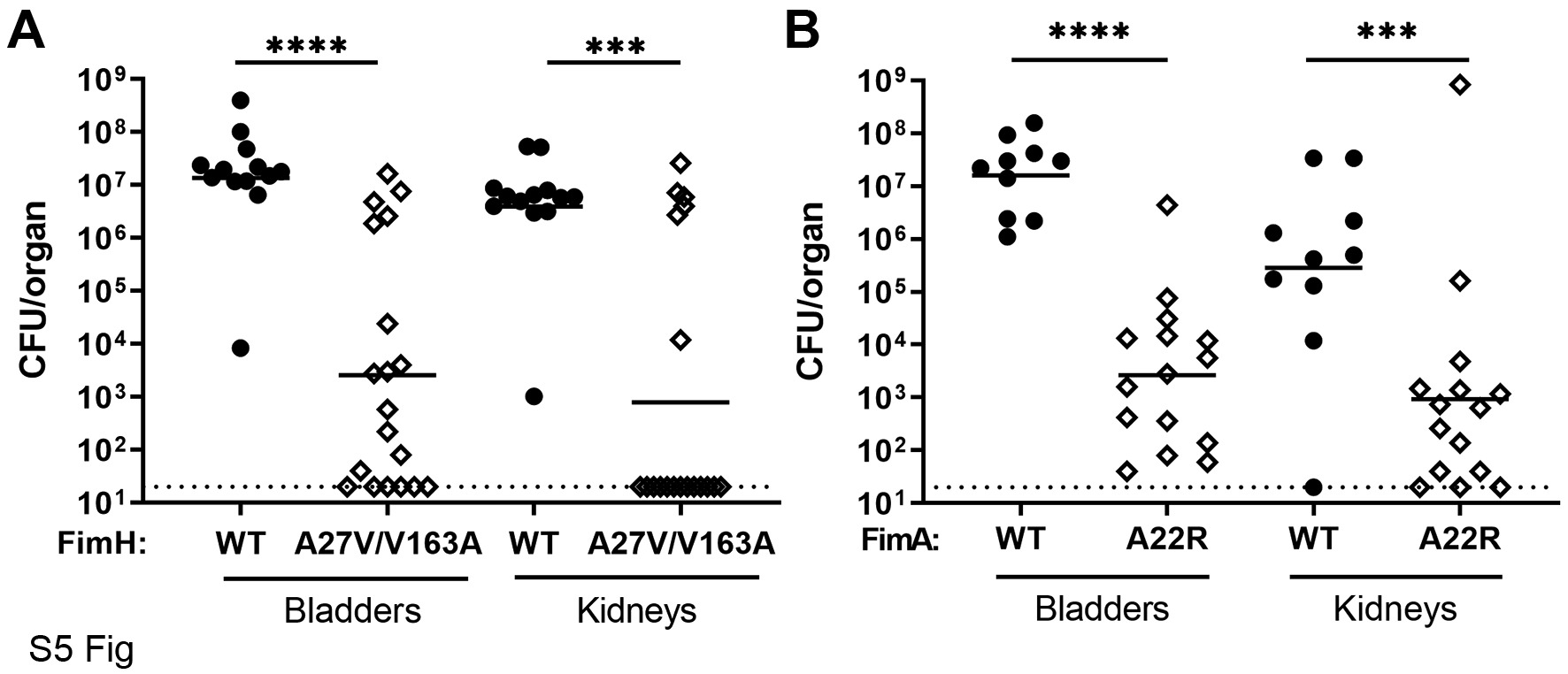

Supplement: S5 Fig — Male C3H/HeN mice were infected with UTI89 (closed circles) or with the indicated type 1 pili variants. Bladders and kidneys harvested 2 wpi yielded higher bacterial loads of wild-type (WT) UTI89 compared to (A) UTI89 FimHA27V/V163A (open diamonds) (bladder ****p<0.0001, kidney ***p = 0.0001) or (B) UTI89 FimAA22R (open diamonds) (bladder ****p<0.0001, kidney ***p = 0.0038). Horizontal bars indicate geometric mean, and dotted line indicates limit of detection. A) n = 13–19 mice per condition over 3 independent experiments; B) n = 10–15 mice per condition over 2 independent experiments. (TIF) [file ppat.1009314.s005.tif]

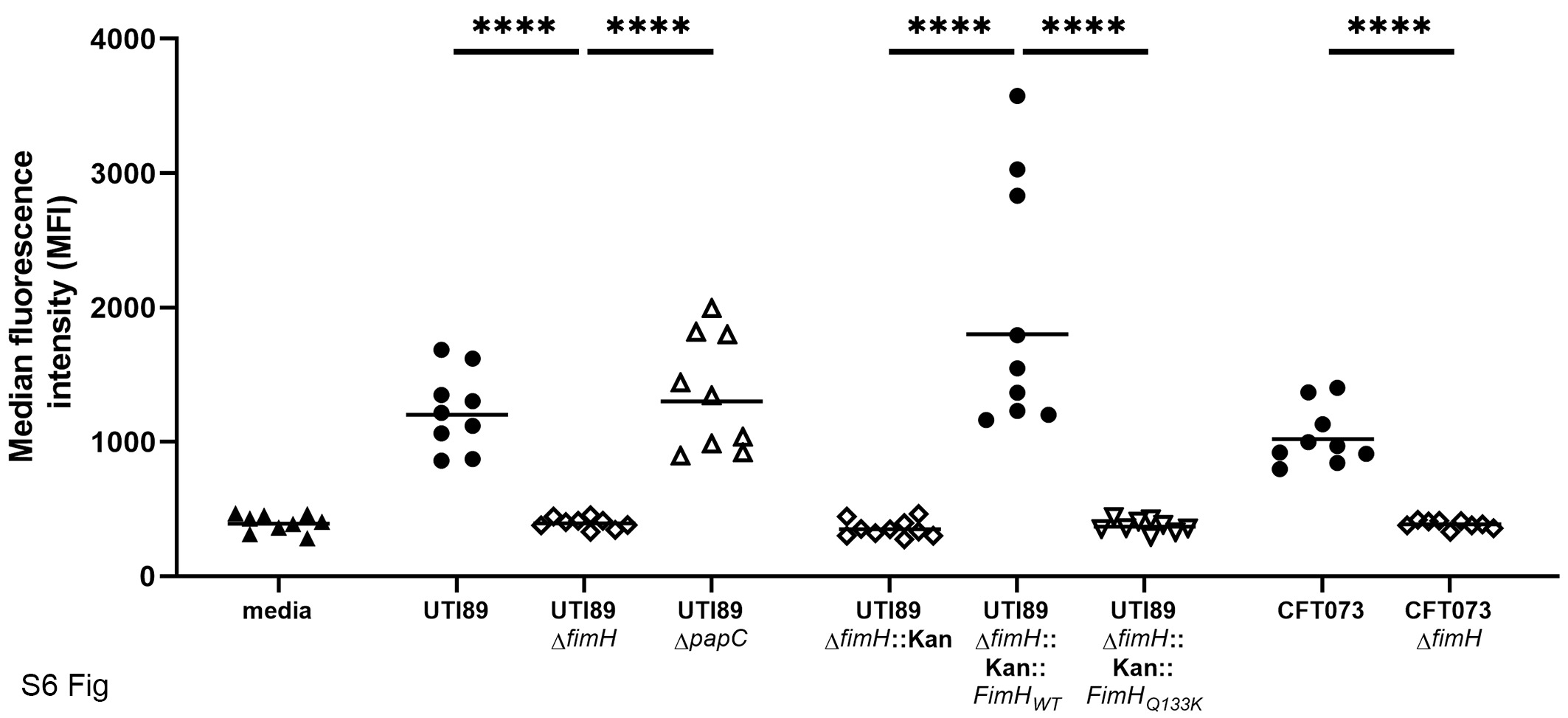

Supplement: S6 Fig — Cultured murine collecting duct cells were infected with the indicated UPEC strains, and cells were stained with anti-E. coli antibody and analyzed by flow cytometry; median fluorescence intensities are shown. Mutation of fimH in UTI89 abrogated binding, while deletion of the P pilus usher (papC) had no effect. Chromosomal re-integration of wild-type fimH, but not fimHQ133K, restored binding in the UTI89 fimH mutant. Deletion of fimH in CFT073 similarly abrogated binding by this urosepsis strain. n = 9–10 wells per condition (aggregate of three triplicate experiments). ****p<0.0001. (TIF) [file ppat.1009314.s006.tif]

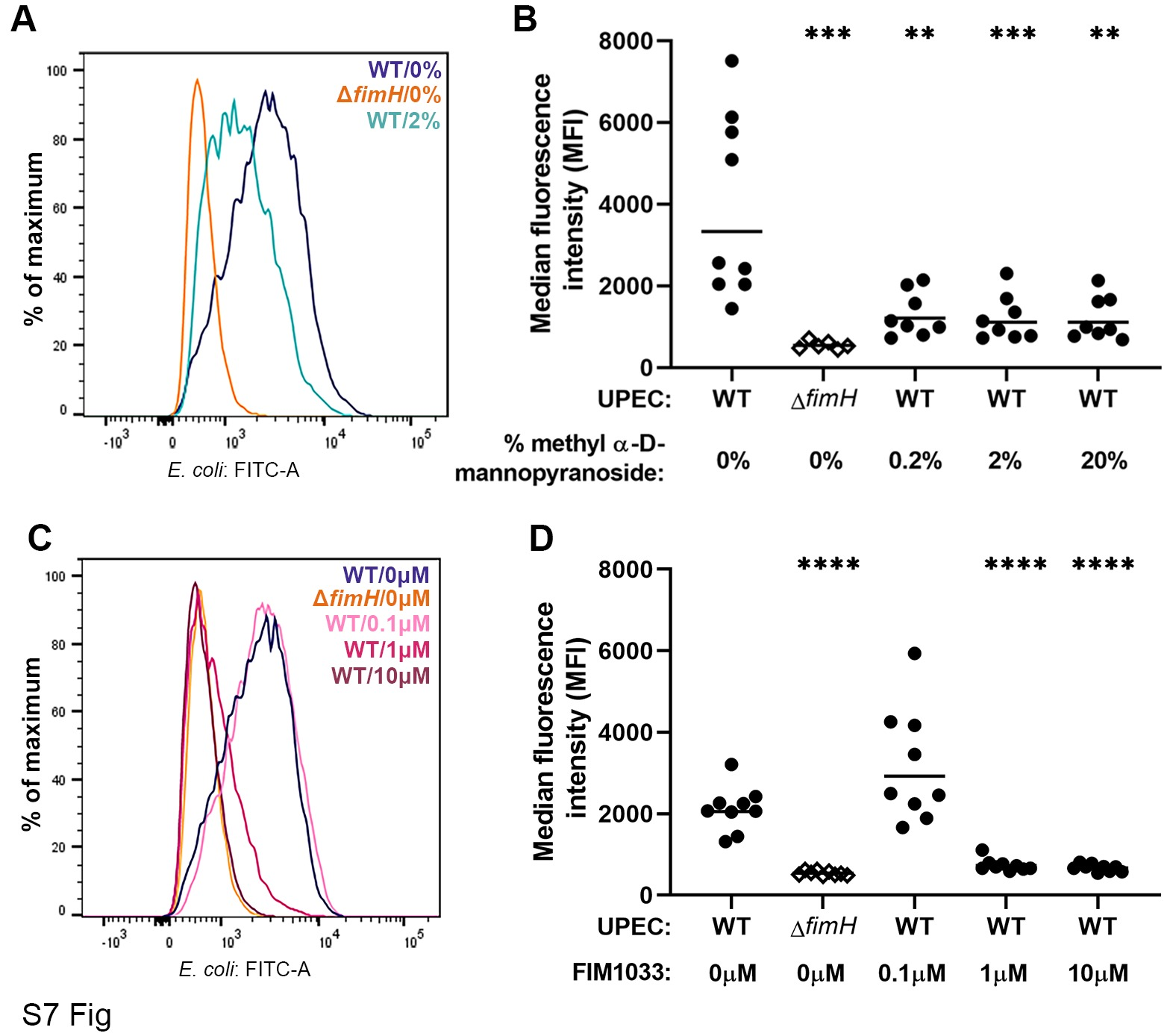

Supplement: S7 Fig — A) FITC signal (anti-E. coli) on IMCD-3 cells infected with wild-type UTI89 (navy blue) compared to infection with UTI89 ΔfimH (orange) or with wild-type UTI89 in media containing 2% methyl α-d-mannopyranoside (teal). B) Binding of IMCD-3 cells by UTI89 (closed circles) with or without addition of methyl α-d-mannopyranoside, or with ΔfimH (open diamonds), was quantified by flow cytometry after gating on single cells. Significance is shown in comparison to WT UTI89 without methyl α-d-mannopyranoside (**p<0.01, ***p<0.001). n = 6–9 samples per condition over 3 independent experiments. C) FITC signal (anti-E. coli) on IMCD-3 cells infected with wild-type UTI89 (navy blue) compared to infection with UTI89 ΔfimH (orange) or with wild-type UTI89 in media containing FIM1033 (0.1μM, light pink; 1μM, dark pink; 10μM, maroon). D) Binding of IMCD-3 cells by WT UTI89 (closed circles) with or without addition of FIM1033, or by ΔfimH (open diamonds), was quantified by flow cytometry after gating on single cells. Horizontal bars indicate geometric mean, and significance is shown in comparison to WT UTI89 without FIM1033 (****p<0.0001). n = 9 samples per condition over 3 independent experiments. (TIF) [file ppat.1009314.s007.tif]

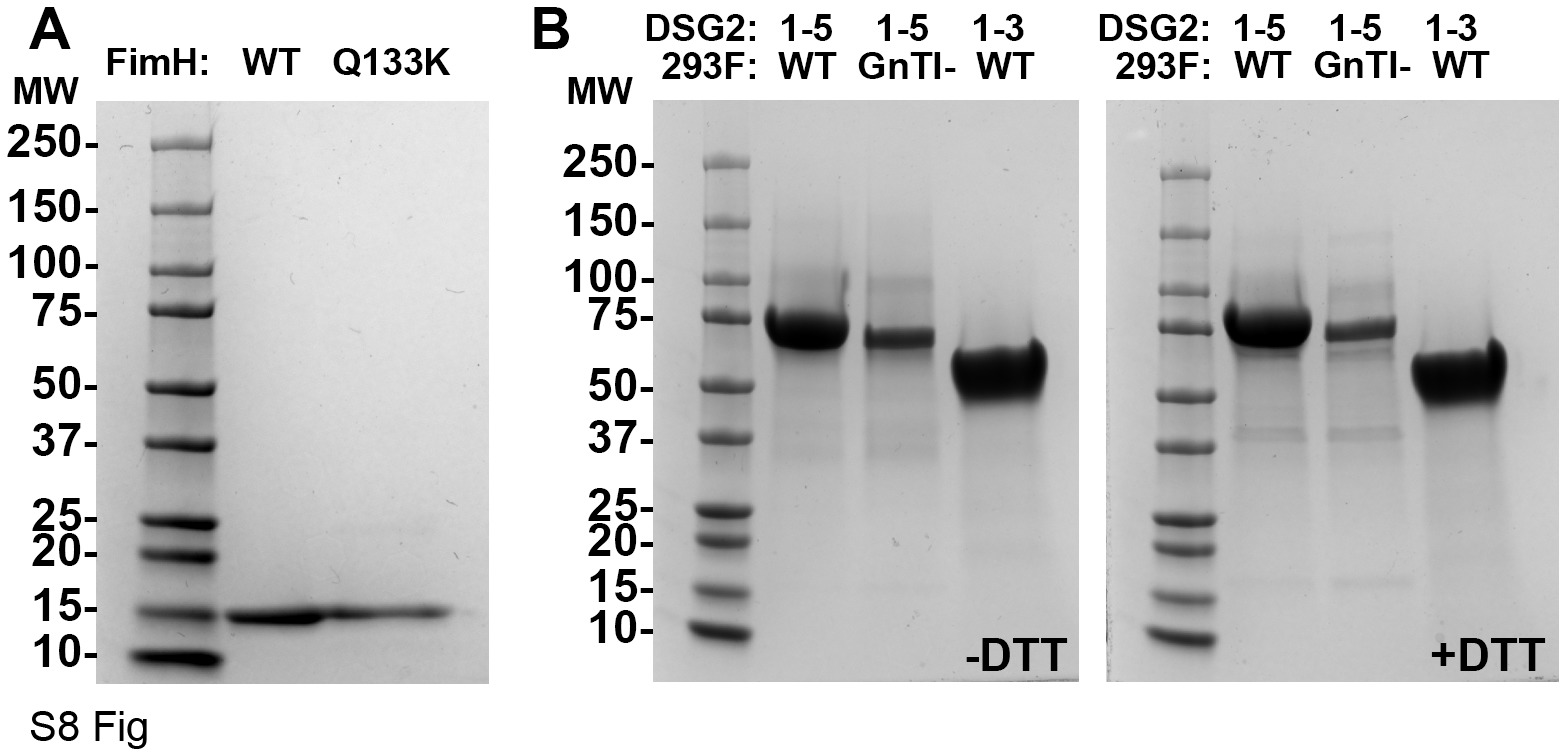

Supplement: S8 Fig — A) Coomassie blue-stained gel of FimHLD and FimHQ133K. B) Coomassie blue-stained gel after metal-affinity purification of DSG2 EC1-5 and EC1-3 from cell supernatant. DSG2 EC1-5 was expressed in both WT Expi293F cells as well as in Expi293F GnTI- cells (lacking complex glycans). In right panel, due to its immunoglobulin folds, addition of the reducing agent DTT alters the apparent molecular weight (MW) of DSG2 EC1-5, from ~70 kDa to ~75 kDa. (TIF) [file ppat.1009314.s008.tif]

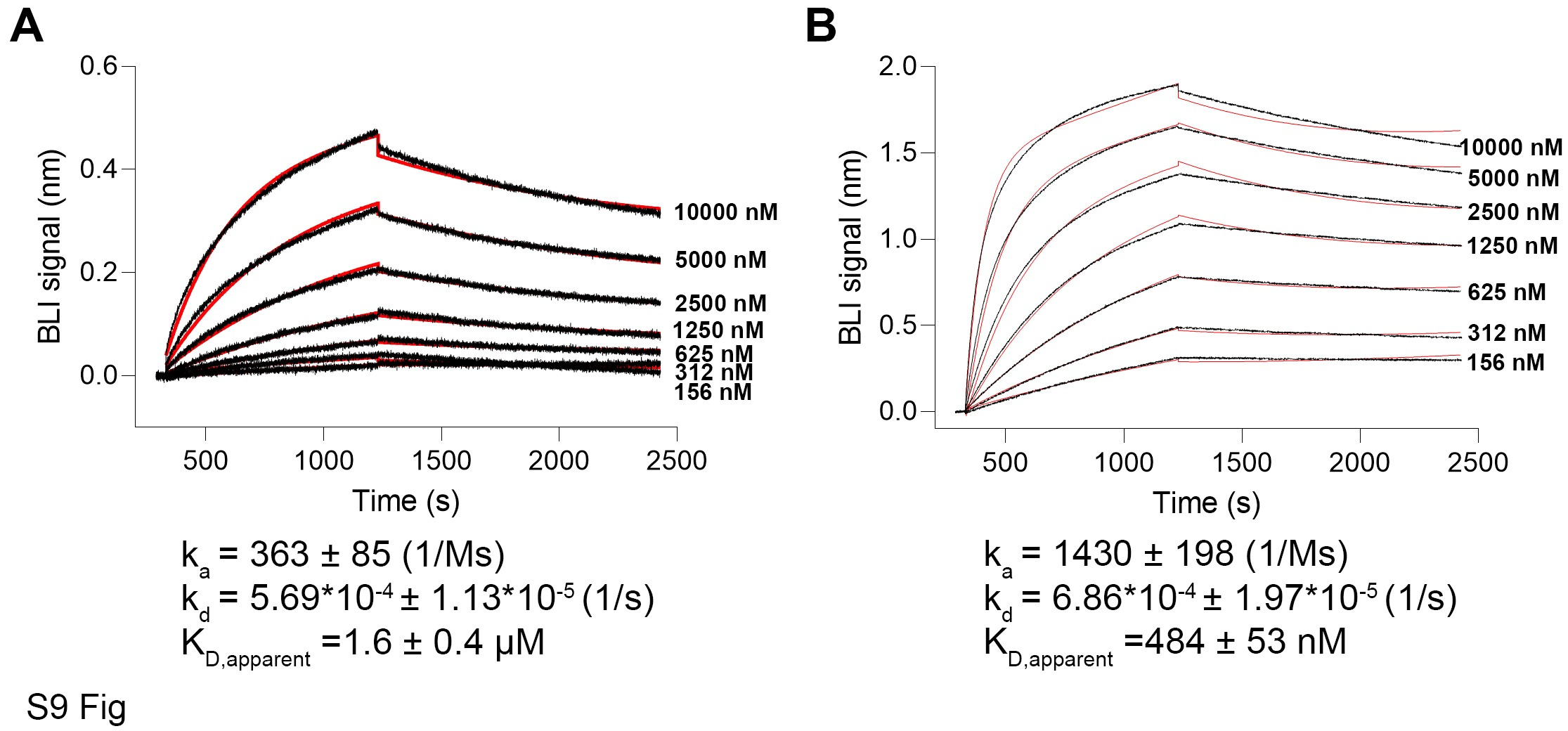

Supplement: S9 Fig — A 1:1 binding model (red lines) was used to fit experimental curves (black lines). Representative curves shown from 5 independent BLI runs per condition; affinity values represent the mean ± SD of two independent experiments. (TIF) [file ppat.1009314.s009.tif]

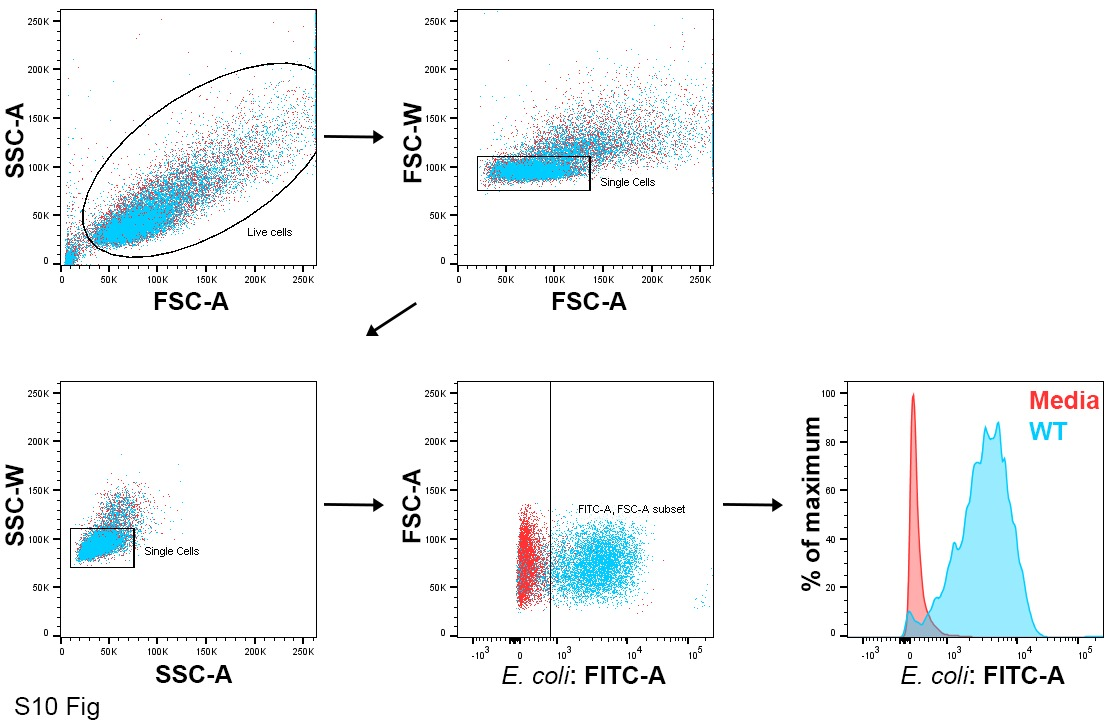

Supplement: S10 Fig — Samples were gated on single cells. Representative samples of IMCD-3 cells treated with medium alone (red) and WT UTI89 (blue) shown. (TIF) [file ppat.1009314.s010.tif]
