# Supplementary material for: A host receptor enables type 1 pilus-mediated pathogenesis of Escherichia coli pyelonephritis
Source: PLoS Pathog. 2021 Jan 29;17(1):e1009314. doi: 10.1371/journal.ppat.1009314 (PMC7875428; doi:10.1371/journal.ppat.1009314)
Supplement: S2 Table — (DOCX) [file ppat.1009314.s012.docx]

**S2 Table.** Strains used in this study

| **Strain** | **Description** | **Reference** |
| --- | --- | --- |
| UTI89 | Prototypic UPEC isolate used for mouse infections and *in vitro* binding | [1] |
| UTI89 ∆*fimH* | Type 1 pilus adhesin mutant in UTI89 used for mouse infections and *in vitro* binding | [1] |
| CFT073 | Pyelonephritis/urosepsis isolate used for mouse infections and *in vitro* binding | [2] |
| CFT073 ∆*fimH* | Type 1 pilus adhesin mutant in CFT073 used for mouse infections and *in vitro* binding | [2] |
| UTI89 FimH_WT_ | Kan-resistant comparator for FimH_A27V/ V163A_ | [3] |
| UTI89 FimH_A27V/ V163A_ | Functional variant of the FimH adhesin | [3] |
| UTI89 FimA_WT_ | Spc-resistant comparator for FimA_A22R_ | [4] |
| UTI89 FimA_A22R_ | Functional variant of the FimA pilus rod | [4] |
| UTI89 HK::Kan | Kan-resistant comparator for CUP pilus co-infections | [5] |
| UTI89 HK::Cm | Cm-resistant comparator for CUP pilus co-infections | [5] |
| UTI89∆*yqi* | Full Yqi operon knockout for mouse studies | [6, 7] |
| UTI89∆*sfaA-H* | Full Sfa operon knockout for mouse studies | [6, 7] |
| UTI89∆*yad* | Full Yad operon knockout for mouse studies | [6, 7] |
| UTI89∆*yfc* | Full Yfc operon knockout for mouse studies | [6, 7] |
| UTI89∆*yeh* | Full Yeh operon knockout for mouse studies | [6, 7] |
| UTI89∆*F17-like* | Full F17-like operon knockout for mouse studies | [6, 7] |
| UTI89∆*auf* | Full Auf operon knockout for mouse studies | [6, 7] |
| UTI89∆*papC* | Pap pilus usher knockout used for mouse infections and *in vitro* binding | [6, 7] |
| UTI89∆*yagW* | Mat pilus adhesin knockout for mouse studies | [7] |
| UTI89∆*fimH*::Kan | Type 1 pilus adhesin mutant in UTI89 used for *in vitro* binding (S6 Fig) | [8] |
| UTI89∆*fimH*::Kan::*FimH_WT_* | Type 1 pilus adhesin mutant with chromosomal re-integration, used for *in vitro* binding (S6 Fig) | [8] |
| UTI89∆*fimH*::Kan::*FimH_Q133K_* | Type 1 pilus adhesin mutant with chromosomal re-integration of the Q133K mutant, used for *in vitro* binding (S6 Fig) | [8] |

**REFERENCES (for S2 Table)**

1. Wright KJ, Seed PC, Hultgren SJ. Development of intracellular bacterial communities of uropathogenic *Escherichia coli* depends on type 1 pili. Cell Microbiol. 2007;9(9):2230-41.

2. Welch RA, Burland V, Plunkett G, 3rd, Redford P, Roesch P, Rasko D, et al. Extensive mosaic structure revealed by the complete genome sequence of uropathogenic *Escherichia coli*. Proc Natl Acad Sci U S A. 2002;99(26):17020-4.

3. Kalas V, Pinkner JS, Hannan TJ, Hibbing ME, Dodson KW, Holehouse AS, et al. Evolutionary fine-tuning of conformational ensembles in FimH during host-pathogen interactions. Sci Adv. 2017;3(2):e1601944.

4. Spaulding CN, Schreiber HLt, Zheng W, Dodson KW, Hazen JE, Conover MS, et al. Functional role of the type 1 pilus rod structure in mediating host-pathogen interactions. eLife. 2018;7. doi: 10.7554/eLife.31662.

5. Schwartz DJ, Chen SL, Hultgren SJ, Seed PC. Population dynamics and niche distribution of uropathogenic *Escherichia coli* during acute and chronic urinary tract infection. Infect Immun. 2011;79(10):4250-9.

6. Greene SE, Pinkner JS, Chorell E, Dodson KW, Shaffer CL, Conover MS, et al. Pilicide ec240 disrupts virulence circuits in uropathogenic *Escherichia coli*. mBio. 2014;5(6):e02038.

7. Spaulding CN, Klein RD, Ruer S, Kau AL, Schreiber HL, Cusumano ZT, et al. Selective depletion of uropathogenic *E. coli* from the gut by a FimH antagonist. Nature. 2017;546(7659):528-32.

8. Chen SL, Hung CS, Pinkner JS, Walker JN, Cusumano CK, Li Z, et al. Positive selection identifies an *in vivo* role for FimH during urinary tract infection in addition to mannose binding. Proc Natl Acad Sci U S A. 2009;106(52):22439-44.
