# Supplementary material for: A host receptor enables type 1 pilus-mediated pathogenesis of Escherichia coli pyelonephritis
Source: PLoS Pathog. 2021 Jan 29;17(1):e1009314. doi: 10.1371/journal.ppat.1009314 (PMC7875428; doi:10.1371/journal.ppat.1009314)
Supplement: S3 Table — (DOCX) [file ppat.1009314.s013.docx]

**S3 Table.** Frameshift mutations within *Dsg2* in IMCD-3 clones C4 and F11 (two allelic variants present in each clone are shown)

| **C4** (1 bp deletion) | AGCCCTTGGAACTACGCATCAAA**–**TTCTGGACATCAATGACAACGAGCCA |
| --- | --- |
| **C4** (1-bp insertion) | AGCCCTTGGAACTACGCATCAAAGTT**T**CTGGACATCAATGACAACGAGCCA |
| **F11** (2-bp deletion) | AGCCCTTGGAACTACGCATCAA**– –**TTCTGGACATCAATGACAACGAGCCA |
| **F11** (1-bp insertion) | AGCCCTTGGAACTACGCATCAAAGTT**T**CTGGACATCAATGACAACGAGCCA |
