# Supplementary material for: A host receptor enables type 1 pilus-mediated pathogenesis of Escherichia coli pyelonephritis
Source: PLoS Pathog. 2021 Jan 29;17(1):e1009314. doi: 10.1371/journal.ppat.1009314 (PMC7875428; doi:10.1371/journal.ppat.1009314)
Supplement: S4 Table — (DOCX) [file ppat.1009314.s014.docx]

**S4 Table.** Probes and primers for qPCR

| Gene | Assay | RefSeq | Exon | Probe and Primers (P1/P2) |
| --- | --- | --- | --- | --- |
| *Dsg2* | Mm.PT.58.8263998 | NM_007883 | 6-7 | 5’-/56-FAM/TAGCTGCTG/ZEN/TGTTCCTCTCTGTCCA/3IABkFQ/-3’ |
|  |  |  |  | P1: 5’-GAGCCTGCAAATAGTCATATGTTC-3’ |
|  |  |  |  | P2: 5’-CATCTCTTGCTTCCACCGT-3’ |
| *Actb* | Mm.PT.39a.22214843.g | NM_007393 | 5-6 | 5’-/56-FAM/CTGGCCTCA/ZEN/CTGTCCACCTTCC/3IABkFQ/-3’ |
|  |  |  |  | P1: 5’-GATTACTGCTCTGGCTCCTAG-3’ |
|  |  |  |  | P2: 5’-GACTCATCGTACTCTGCTTG-3’ |
| *Gapdh* | Mm.PT.39a.1 | NM_008084 | 2-3 | 5’-/56-FAM/TGCAAATGG/ZEN/CAGCCCTGGTG/3IABkFQ/-3’ |
|  |  |  |  | P1: 5’-AATGGTGAAGGTCGGTGTG-3’ |
|  |  |  |  | P2: 5’-GTGGAGTCATACTGGAACATGTAG-3’ |
